# Rapid dehydration drives a nondiffusional drop in C_3_ photosynthesis that aligns with phosphate limitation

**DOI:** 10.1111/nph.71236

**Published:** 2026-05-18

**Authors:** Chandra Bellasio, Daniel Tholen, Hilary Stuart‐Williams, Graham D. Farquhar, Jaume Flexas

**Affiliations:** ^1^ Laboratory of Theoretical and Applied Crop Ecophysiology, School of Biology and Environmental Science University College Dublin Belfield, D04 V1W8 Dublin Ireland; ^2^ Department of Chemistry, Biology and Biotechnology Università Degli Studi Di Perugia Perugia 06123 Italy; ^3^ Biology of Plants Under Mediterranean Conditions, Department of Biology University of the Balearic Islands Palma 07122 Illes Balears Spain; ^4^ Research School of Biology Australian National University Acton Australian Capital Territory 2601 Australia; ^5^ Department of Ecosystem Management, Climate and Biodiversity Institute of Botany, BOKU University Vienna 1180 Austria; ^6^ Agro‐Environmental and Water Economics Research Institute (INAGEA) Complex Balear de Recerca Desenvolupament Tecnològic i Innovació (Parc Bit) Carrer Blaise Pascal, 6 Palma 07120 Illes Balears Spain

**Keywords:** assimilation, drought, isotopic discrimination, mesophyll conductance, stomatal conductance, stress, sunflower, wheat

## Abstract

- Drought is an abnormally prolonged water deficit posing major challenges to plants. Stomatal closure has long been considered the primary factor limiting photosynthesis during the early stages of drought. However, emerging evidence suggests that nonstomatal limitation may also arise, particularly under rapid dehydration.
- We hypothesised that under rapid dehydration, photosynthesis is constrained by an impeded supply of CO_2_ to the chloroplasts. To test this, we conducted an innovative experiment inducing rapid but controlled dehydration in hydroponically grown wheat and sunflower. We measured water vapour and CO_2_ exchange in real time, along with their isotopic compositions and Chl fluorescence.
- Our results revealed a decrease in CO_2_ levels in the substomatal cavity at the onset of drying, followed by a reduction in assimilation rate due to both stomatal and nonstomatal limitations. The nonstomatal component was attributed to a combination of diffusional limitation, linked to reduced mesophyll conductance, and a biochemical limitation.
- Our analyses indicate that the biochemical limitation is primarily due to a reduction in ATP production, rather than a diminished capacity for Rubisco carboxylation. This is likely attributable to a phosphate shortage, which aligns with an observed reversal in the oxygen sensitivity of CO_2_ assimilation.

## Graphical abstract

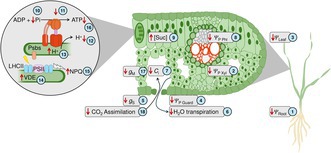

## Introduction

Understanding the impact of drought on plants is crucial. Drought has a devastating impact on crops, affecting three‐quarters of the global area cultivated with maize, rice, soy, and wheat, leading to food shortages and economic hardship. Climate change is aggravating the situation, increasing the severity, frequency, and duration of drought events (Naumann *et al*., 2021). The combined reduction in air humidity and depletion of soil moisture often results in a decrease in leaf water potential (*Ψ*_L_), which can drop swiftly within hours (Biscoe *et al*., 1976; Dutt & Gill, 1978; Jones, 1978; Reicosky *et al*., 1982). This is reflected in the transient wilting of leaves of several crop species, such as wheat or soybean, that is typically observed around midday (Angus & Moncur, 1977; Boyer *et al*., 1980). The associated reduction in CO_2_ assimilation is generally attributed to stomatal closure, and expressed as stomatal limitation (*L*_S_); the theoretical framework for which is detailed in Bellasio (2025). However, nonstomatal limitation (*L*_NS_) may also come into play especially when dehydration is rapid (Boyer & Ghorashy, 1971; Cornic *et al*., 1983). For instance, while a moderate *Ψ*_L_ decrease (*c*. −1 MPa) over 3 d minimally impacted C_3_ assimilation (Quirk *et al*., 2019), a more rapid decrease (within *c*. 6 h) led to a sharp drop in assimilation due to rising *L*_NS_ (Bellasio *et al*., 2023). The specific mechanisms driving such rate‐dependent responses remain to be characterised. Drought typically reduces mesophyll conductance (*g*_M_), potentially inducing *L*_NS_ by restricting CO_2_ supply (Flexas *et al*., 2004). However, there are exceptions. For instance, Hommel *et al*. (2014) found that one of five C_3_ forest species maintained stable *g*_M_ under drought. Studies imposing drought over days to weeks potentially confound direct stress effects (strain) with plant responses (tolerance or escape mechanisms). Leaves developed under hydrated conditions undergo significant acclimation when subjected to long‐term drought. This transition involves the dismantling of photosynthetic machinery, the thickening and lignification of cell walls, and the accumulation of defense metabolites, all of which represent potential constraints on *g*_M_. By contrast, leaves emerging during prolonged drought may adopt alternative strategies that may entail acquiring a higher *g*_M_ than well‐watered counterparts (Roig‐Oliver *et al*., 2020). Experiments investigating rapid dehydration are needed to distinguish initial strain from long‐term tolerance or escape responses.

Our research sought to elucidate the physiological mechanism that results in photosynthetic limitation under rapid dehydration. We hypothesize that the surge of *L*_NS_ during rapid dehydration in C_3_ plants is due to a decrease in *g*_M_. We induced rapid controlled dehydration in hydroponically grown plants, measuring Chl fluorescence, CO_2_, and water exchanged by leaves and their isotopic composition. *g*_M_ was estimated from electron transport, carbon and oxygen isotope discrimination, with adjustments for cuticular transpiration and potential water vapour unsaturation in intercellular spaces. We tested for a reduction in *g*_M_ and analysed whether it, alongside biochemical traits derived from gas exchange, could explain the observed *L*_NS_.

## Materials and Methods

### Plants

*Helianthus annuus* L. and *Triticum aestivum* L. seeds were germinated on perlite, transferred into foam rubber discs, and placed in 5‐cm holes cut in the lids of 20‐l water tubs aerated through aquarium stones. The growth solution was initially fertilised with 150 cm^3^, then weekly with 50 cm^3^, of Green Dream 1 complete fertiliser (Flairform, Applecross, WA, Australia), and replaced after 3 wk. Plants were grown for 4–6 wk in controlled environment chambers (Thermoline Scientific, Wetherill Park, NSW, Australia), at 26° : 20°C (day : night), 80% relative humidity, with a 12‐h light, which included a 9‐h period with moderate light (400 μmol m^−2^ s^−1^), a 1‐h midday peak illumination (690 μmol m^−2^ s^−1^, 1000 W metal halide arc lamps multi vapour® MVR, GE lighting, East Cleveland, OH, USA, plus halogen), and 1 h of low light (80 μmol m^−2^ s^−1^, only halogen) at dawn and dusk.

### Hydromechanical characterisation

Pressure–volume curves were measured on *n* = 7 replicates and analysed to determine the elastic modulus (*ε*) and apoplastic water fraction (awf), which were then used to derive water potential at turgor loss (*Ψ*_TL_) from measurements of bulk solute water potential at full hydration as per Bellasio *et al*. (2023), summarised in Supporting Information Methods S1 and Fig. S1.

### Instrumental set‐up: coupling gas exchange, fluorescence, and isotopic monitoring

A portable gas‐exchange system (LI6400XT; LI‐COR Biosciences, Lincoln, NE, USA) was fitted with a 6400–06 PAM2000 adapter on a 6‐cm^2^ cuvette or a custom‐made equivalent based on a 12‐cm^2^ grass cuvette used for all isotopic measurements, and a Dual PAM–F (Heinz Walz GmbH, Effeltrich, Germany). Uniform illumination was provided by a LI6400‐18 RGB light source. CO_2_ diffusion leaks from the cuvette were physically compensated for by adding *c*. 1 m of PVC tubing (internal diameter 4 mm, external diameter 6 mm) to the reference tube upstream of the sensor head and adjusting the partitioning of the flow between reference and sample lines. The extended surface area of the tubing provides a path for CO_2_ diffusion, while adjusting the flow split controls the residence time available for that exchange in each line. Consequently, the reference diffusion leak can be precisely matched to the sample chamber leak. We identified the balance point by measuring dark respiration at 0 and 2000 μmol mol^−1^ CO_2_ by logging a 15 s average for 5 min, matching every minute; the flow split that rendered respiration invariant was maintained for all measurements. This physical balancing serves as the hardware equivalent to the mathematical calibration described by Kitao *et al*. (2017).

To avoid underestimating *g*_M_ when stomata close, we accounted for small H_2_O fluxes across the cuticle (Methods S2). For these corrections, we assumed a moderate cuticular conductance to water vapour (*g*_cw_ = 5 mmol m^−2^ s^−1^) and a ratio between cuticular conductance to CO_2_ and water (*β*) at 0.025 (Márquez *et al*. 2021). Sensitivity analysis across *g*_cw_ values from 0 to 10 mmol m^−2^ s^−1^ confirmed that our conclusions remain robust to this parameter (Note S1; Fig. S6). Rapid response mass flow controllers (Alicat Scientific, Tucson, AZ, USA) operated by custom software (vinland, running in Microsoft windows) on a Raspberry Pi 3 (raspberrypi.org) mixed O_2_ and N_2_. The synthetic air was humidified and cooled to a dew point of *c*. 14°C to maintain a water mole fraction deficit in the leaf cuvette of *c*. 10 mmmol mol^−1^ CO_2_ (BOC, North Ryde, NSW, Australia; the isotopic composition *δ* ^13^C was −7.02‰ against Vienna Pee Dee Belemnite, VPDB, calibrated on an Isoprime dual‐inlet Isotope Ratio Mass Spectrometer) was added using the CO_2_ injection unit of the LI6400XT. The air mixture was split into four lines. Two fed the LI6400XT reference and sample cuvette. The third was connected to a cavity ring‐down absorption spectrometer (L1102‐i; Picarro Inc., Santa Clara, CA, USA), measuring the ^1^H_2_^16^O and ^1^H_2_^18^O isotopologues of H_2_O. The L1102‐i was periodically calibrated using a capillary nebuliser (Fig. S2): pressurized reference water (calibrated against International Atomic Energy Agency standards: Vienna Standard for Light Antarctic Precipitation, VSLAP, Greenland Ice Sheet Project, GISP, and Vienna Standard Mean Ocean Water, VSMOW) was instantly and completely vapourised by spraying it onto a canister‐enclosed computer fan, ensuring constant composition of the vapour carried by a dry nitrogen flow. Water concentration depended on the feed rate through the capillary, controlled by adjusting the reservoir height. Moving the reservoir up or down produced calibration curves across concentrations. The fourth line passed in a 2 l min^−1^ dry N_2_ counterflow through a Polytube Dryer (Perma Pure, Lakewood, CO, USA), consisting of Nafion™ (Chemours, Wilmington, DE, USA) capillary membranes selectively diffusing water vapour, while retaining other gases dehydrated to a dew point of −40°C, and fed to a Quantum‐Cascade Laser system (QCL; Aerodyne Research, Billerica, MA, USA), measuring specifically the CO_2_ isotopologues ^12^C^16^O_2_, ^13^C^16^O_2_, and ^12^C^18^O^16^O. The sample gas from the LI6400XT cuvette exhaust was split into two lines: one fed to the L1102‐i and the other Nafion‐dried as described and fed to the QCL. In the QCL inlet, the CO_2_ mole fraction of the reference gas was matched to the sample by diluting with synthetic air from upstream of the LI6400XT CO_2_ mixing unit, with the rate controlled by a micrometric needle valve.

### Gas exchange on hydrated plants

On a first set of four thoroughly hydrated plants, photosynthetic photon flux density (PPFD) was set at 500 μmol m^−2^ s^−1^, reference CO_2_ mole fraction was 420 μmol mol^−1^. After a minimum of 1 h in the light, photosynthetic response curves to CO_2_ (*A*/*C*_i_ curves) and to PPFD (*A*/PPFD curves) were recorded at 25°C.

The relationship between *A* and *C*_i_ was modelled empirically as per Bellasio *et al*. (2016) (briefly described in Method S3) to derive a set of parameters describing the response curves (Table 1) and respiration in the light (*R*_Light_).

**Table 1:** Fitted photosynthetic parameters in fully hydrated leaves.

	Symbol	Units	Wheat		Sunflower	
			Ambient O_2_	2% O_2_	Ambient O_2_	2% O_2_
From *A/C*_i_ curves	*A*_SAT_	μmol m^−2^ s^−1^	25.6 (0.40)	25.4 (0.48)	32.5 (0.63)	33.2 (0.85)
	CE	mol m^−2^ s^−1^	0.123 (0.0019)	0.196 (0.013)	0.182 (0.0082)	0.327 (0.027)
	*ω*	Dimensionless	0.670 (0.038)	0.914 (0.016)	0.511 (0.033)	0.859 (0.023)
	*Γ*	μmol mol^−1^	44.7 (0.36)	6.30 (0.55)	43.9 (0.27)	7.27 (0.274)
From *A/C*_M_ curves	CE^1^	mol m^−2^ s^−1^	0.169 (0.0035)	0.365 (0.034)	0.548 (0.053)	3.96 (2.0)
	CE^2^	mol m^−2^ s^−1^	0.178 (0.0039)	0.403 (0.047)	0.275 (0.018)	0.904 (0.21)
	CE^3^	mol m^−2^ s^−1^	0.158 (0.0030)	0.309 (0.030)	0.234 (0.013)	0.544 (0.089)
	*ω* ^1^	Dimensionless	0.546 (0.052)	0.825 (0.029)	*c.*0	0.432 (0.26)
	*ω* ^2^	Dimensionless	0.524 (0.055)	0.805 (0.034)	0.292 (0.057)	0.600 (0.12)
	*ω* ^3^	Dimensionless	0.577 (0.049)	0.854 (0.025)	0.392 (0.041)	0.768 (0.065)
From *A/*PPFD curves	GA_SAT_	μmol m^−2^ s^−1^	34.4 (0.56)	44.9 (1.5)	40.8 (2.5)	50.9 (1.3)
	*Y*(CO_2_)_LL_	Dimensionless	0.0479 (0.00086)	0.0627 (0.0011)	0.0561 (0.00052)	0.0721 (0.0011)
	*m*	Dimensionless	0.758 (0.022)	0.782 (0.033)	0.835 (0.015)	0.885 (0.025)
	LCP	μmol m^−2^ s^−1^	15.3 (1.6)	14.7 (0.98)	20.2 (1.2)	19.0 (1.5)
	*R*_Light_	μmol m^−2^ s^−1^	0.727 (0.075)	0.914 (0.060)	1.13 (0.070)	1.37 (0.12)

Mean values (±1 SE) for parameters of nonrectangular hyperbolas fitted to *A–*PPFD and *A–C*_i_ within the modelling framework of Bellasio *et al.* (2016); *n* = 4 biological replicates. *A*_SAT_ is the horizontal asymptote of the *A*–*C*_i_ curve representing the CO_2_‐saturated assimilation rate; CE is carboxylating efficiency, that is the slope of the inclined asymptote, or the initial slope of the *A*–*C*_i_ curve; *Γ* is the *C*_i_–*A* compensation point, that is *C*_i_ where *A* = 0; GA_SAT_ is the horizontal asymptote of the PPFD–*A* curve, *ω* is the empirical curvature; LCP is light compensation point, that is PPFD where *A* = 0; *R*_Light_ is Respiration in the light (or day‐respiration); *Y*(CO_2_)_LL_ is the inclined asymptote, that is, the initial (or maximum) quantum yield for CO_2_ fixation; *m* is the empirical curvature. *A*/*C*_M_ curves in wheat were obtained (^1^) for a *g*_M_ = 0.45 mol m^−2^ s^−1^; (^2^) for a *g*_M_ = 0.4 mol m^−2^ s^−1^; (^3^) for a *g*_M_ = 0.56 mol m^−2^ s^−1^; in sunflower (^1^) for a *g*_M_ = 0.25 mol m^−2^ s^−1^; (^2^) for a *g*_M_ = 0.53 mol m^−2^ s^−1^; (^3^) for a *g*_M_ = 0.81 mol m^−2^ s^−1^, corresponding to the initial responses obtained for fully hydrated plant using fluorescence, carbon or oxygen proxies.

### Limitations during dehydration

A second set of six plants was subject to high‐resolution gas‐exchange measurements.

The evening before the experiment, plants were bagged in the dark and transferred to the laboratory. A PSY1 psychrometer (ICT, Armidale, NSW, Australia) was calibrated with five standard NaCl solutions. A small window of epidermis was removed with a razor blade from a fully expanded leaf of a plant standing in aerated water. The window was rinsed with abundant distilled water, blotted with paper and subsequently fitted with the thermocouple of the PSY1, and sealed with a tiny ridge of high vacuum grease following the manufacturer's instructions. An adjacent portion of the leaf was clamped in the cuvette of the LI6400XT. Plants were acclimated in the dark overnight with ambient air supply.

In the morning, plants were acclimated for at least 60 min to a PPFD of 500 μmol m^−2^ s^−1^. Gas exchange was measured, together with *Ψ*_L_, every 10 min under atmospheric O_2_ mole fraction (210 mmol mol^−1^) and reference CO_2_ mole fraction of 800 μmol mol^−1^, which was chosen to minimise *L*_S_. After full photosynthetic induction, plants were drawn out of the hydroponics solution until roughly half of the root volume was in air. For bigger plants, transpiration was promoted by a table fan and a LED light (Fig. S2); dehydration was regulated by pulling roots further out of the solution in such a way that *Ψ*_L_ decreased by *c*. 0.1–0.15 MPa every *c*. 15 min until leaves were wilted, after *c*. 6–8 h.

**Fig. 1:**
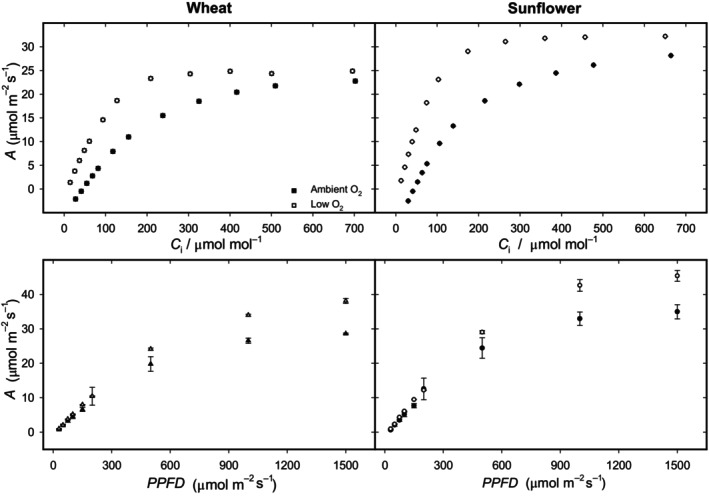
Assimilatory response curves in fully hydrated leaves. Response of assimilation to the mole fraction of CO_2_ in the substomatal cavity (*A*/*C*_i_ curves, upper) and to light intensity (*A*/PPFD curves, lower) were measured under ambient O_2_ mole fraction (210 mmol mol^−1^; filled symbols) or under low O_2_ mole fraction (20 mmol mol^−1^; empty symbols) at a light intensity of 500 μmol m^−2^ s^−1^ (*A*/*C*_i_ curves) or a reference CO_2_ mole fraction of 420 μmol mol^−1^ (*A*/PPFD curves) on fully hydrated wheat (left) or sunflower (right) leaves. *n* = 4 biological replicates for each species. Error bars show ± SE for *A* and *C*_i_.

Stomatal and nonstomatal limitations were resolved after Bellasio *et al*. (2023) by comparing measured gas exchange to a modelled *A*/*C*_i_ curve. If assimilation decreases with (*C*_i_, *A*) pairs still on the *A*/*C*_i_ curve (Fig. 1, top), *A* is limited only by *L*_S_. *L*_NS_ commence as (*C*_i_, *A*) pairs fall below the *A*/*C*_i_ curve, quantified as the vertical relative distance between the operational assimilation and the *A*/*C*_i_ curve.

### Concurrent gas exchange, fluorescence, carbon and oxygen isotopic discrimination

A third set of six plants was subjected to a measurement routine (circa 20–25 min in total), which was repeated until wilting, consisting of stabilisation of the gas exchange under atmospheric O_2_ levels (210 mmol mol^−1^), repeated measurements of isotope ratios in the sample and reference lines, measurement of Chl fluorescence and water potential, and stabilisation under low O_2_ mole fraction (20 mmol mol^−1^) for measurements of gas exchange and Chl fluorescence. The step‐wise decrease in *Ψ*_L_ resulted in steady‐state conditions, which are essential for concurrent gas‐exchange and isotopic measurements (Savage & Cass, 1984; Saathoff & Welles, 2021). CO_2_ isotopologue levels from the QCL was collected by an Arduino Mega (arduino.org), and processed by vinland following the method described by Griffith *et al*. (2012) to calculate CO_2_ isotopic composition (*δ* ^13^C and *δ* ^18^O, see e.g. Ubierna *et al*., 2018b; Holloway‐Phillips *et al*., 2019). The resulting CO_2_ *δ* ^18^O (VPDB) was converted to VSMOW as: *δ* ^18^O (VSMOW) = 1.03092 × *δ* ^18^O (VPDB) + 30.92 (Brand *et al*., 2014). The L1102‐i calculates the isotopic composition of water (*δ* ^2^H and *δ* ^18^O (VSMOW)) directly from its laser measurements and returns these to the user. The laser response was linear with a typical precision below 0.2‰ for *δ* ^18^O. Isotope discrimination (the change in isotopic composition in ambient air induced by the plant during photosynthesis, eqn 3 in Ubierna *et al*. (2018b)) measured for ^13^C ($\Delta_{o}$) or ^18^O ($\Delta_{o}^{18}$) was calculated as per Evans *et al*. (1986):

(Eqn 1) $$\Delta_{o}=\frac{\;\xi \;\left(\delta_{\text{out}}-\delta_{\text{in}}\right)}{1+\delta_{\text{out}}-\xi \;\left(\delta_{\text{out}}-\delta_{\text{in}}\right)}$$

where the isotopic composition *δ* here refers to either *δ* ^13^C or *δ* ^18^O, and *ξ* = *C*_in_/(*C*_in_ − *C*_out_), with *C* representing the ^12^CO_2_ mole fraction in dry air flowing into (*C*_in_) and out of (*C*_out_) the chamber. *ξ* is an index of error amplification that captures trade‐offs between flow rate and assimilation, which warrants caution when exceeding 10 (von Caemmerer & Evans, 1991). In our data, *ξ* > 10 occurred in only five wheat data points under the driest conditions, and discarding them did not affect *g*_M_ trends.

The total electron transport and ATP production rates were calculated with a point‐to‐point calibration after Bellasio *et al*. (2014) as:

(Eqn 2) $$j\;\text{or}\;j_{\mathrm{ATP}}=q\;{\mathrm{GA}}_{\mathrm{Low}}\;\frac{\;Y\left(\mathrm{II}\right)\;}{Y{\left(\mathrm{II}\right)}_{\mathrm{Low}}}$$

where for *j*, *q* = 4.4 (the electrons required by gross assimilation under low [O_2_]), or, for *j*_ATP_, *q* = 5.5 (the ATP required under low [O_2_]) were assumed 10% higher than the theoretical minimum, to account for incomplete photorespiration suppression under 20 mmol mol^−1^ [O_2_]. GA_Low_ is gross assimilation (GA = *A* + *R*_Light_) measured under low O_2_, *R*_Light_ was previously determined on four biological replicates as described previously and was 1.13 and 0.91 μmol m^−2^ s^−1^ for sunflower and wheat, respectively; *Y*(II) and *Y*(II)_Low_ are photosystem II (PSII) yield under atmospheric (210 mmol mol^−1^) and low (20 mmol mol^−1^) [O_2_], respectively.

### Mesophyll conductance from gas exchange and fluorescence

Mesophyll conductance derived from gas exchange and fluorescence was calculated after Harley *et al*. (1992) as:

(Eqn 3) gMJ=ACi−Γ*j+8GAj−4GA

where Γ* is the CO_2_ mole fraction at the mesophyll carboxylating sites at which GA is zero (39.7 in wheat and 37 μmol mol^−1^ in sunflower at 21% O_2_), calculated as $\frac{½\;O}{S_{C/O}}$, where O is the O_2_ mole fraction in air and $S_{C/O}$ is Rubisco specificity (courtesy of S. Whitney, pers. comm.) converted from concentration in solution to mole fraction in air at equilibrium using gas volatility and the calculations in the spreadsheet of Bellasio & Farquhar (2019).

### Mesophyll conductance from carbon isotope discrimination

Mesophyll conductance derived from gas exchange and stable carbon isotope discrimination was calculated after eqn 22 given by Busch *et al*. (2020) as:

(Eqn 4) gMC=1+t1−tAb−am−1+be′1+e′+RLightAe′RLightACaΔi−ΔObs

where *t* is a ternary correction factor, $t=\frac{\left(1+a\right)\;E}{2\;g_{\mathrm{SC}}}$; *E* is leaf transpiration in mmol H_2_O m^−2^ s^−1^; *g*_SC_ is stomatal conductance to CO_2_ in mol air m^−2^ s^−1^; *a* is the weighted ^13^C fractionation representing the effect of diffusion through the stomata and boundary layer; *b* is ^13^C fractionation during carboxylation in C_3_ plants (excluding respiration and photorespiration fractionation) (30‰; McNevin *et al*., 2006), *a*_m_ is the fractionation during diffusion and dissolution in water (1.8‰), $e^{\prime }$ is the total respiratory fractionation representing the isotopic offset of respired CO_2_ relative to current assimilates, encompassing both the kinetic fractionation of respiratory enzymes (e, −3‰, averaged from references in Bellasio & Griffiths (2014) and Ubierna *et al*. (2018a)), and the apparent fractionation caused by the use of older, isotopically distinct carbon substrates assimilated during growth, calculated after Busch *et al*. (2020) as $e^{\prime }=e+\left(\delta_{\text{out}}-\Delta_{o}\right)-\left(\;\delta_{\text{Growth}}-\Delta_{\text{control}}\right)$, where *δ*_Growth_ is the isotopic composition of CO_2_ in the growth chamber (−8.8‰) and Δ_control_ is the average isotopic discrimination measured on hydrated plants under ambient CO_2_ concentration (did not differ between species and was on average −22‰). Δ_i_ is modelled ^13^C isotope discrimination when *g*_M_ is assumed to be infinite. Following eqn S24 in Busch *et al*. (2020):

(Eqn 5) $$\Delta_{i}=\begin{array}{l} \frac{1}{1-t}\left[a\frac{C_{a}-C_{i}}{C_{a}}\right]+\frac{1+t}{1-t}[b\frac{C_{i}}{C_{a}}\\ -\;\frac{1+b}{\left(1+\frac{R_{\text{Light}}}{A}\frac{e^{\prime }}{1+e^{\prime }}\right)}\left(\frac{e^{\prime }}{1+e^{\prime }}\frac{R_{\text{Light}}}{A}\frac{C_{i}}{C_{a}}-\frac{f}{1+f}\frac{\Gamma^{*}}{C_{a}}\right)] \end{array}$$

where *C*_a_ and *C*_i_ are the CO_2_ mole fractions in the cuvette and substomatal airspaces as given by the LI6400XT; and *f* is fractionation during photorespiration (11.6‰; Lanigan *et al*., 2008).

### Mesophyll conductance from ^18^O photosynthetic discrimination

Mesophyll conductance can also be derived from gas exchange and stable oxygen isotope discrimination (Gillon & Yakir, 2000; Barbour *et al*., 2016). We calculated this estimate for *g*_M_ after Ubierna *et al*. (2017),

(Eqn 6) gMO=ACi1−δi18−δA18−am18−δA18am18δce18−δA18−am18−δA18am18

where $\;a_{m}^{18}$ is the summed discrimination against C^18^O^16^O during liquid phase diffusion and dissolution (0.8‰; Farquhar & Lloyd 1993), ${\;\delta }_{A}^{18}$ is the *δ* ^18^O of the CO_2_ taken up by photosynthesis (Evans *et al*., 1986; Cernusak *et al*., 2004) calculated as: ${\;\delta }_{A}^{18}=\delta_{\text{out}}^{18}-\xi \left(\delta_{\text{out}}^{18}-\delta_{\text{in}}^{18}\right)$, where $\delta_{\text{in}}^{18}$ and $\delta_{\text{out}}^{18}$ are the isotopic compositions of the CO_2_ in the air entering and leaving the leaf cuvette. $\delta_{i}^{18}$ is the *δ* ^18^O of the CO_2_ in the substomatal cavity (Farquhar & Cernusak, 2012) calculated as:

(Eqn 7) $$\delta_{i}^{18}=\begin{array}{l} \frac{1}{1+t^{18}}[(\delta_{A}^{18}\left(1-\frac{C_{a}}{C_{i}}\right)\left(1+a^{18}\right)+\frac{C_{a}}{C_{i}}\left(\delta_{\text{out}}^{18}-a^{18}\right)\\ +\;a^{18})+t^{18}\left(\delta_{A}^{18}\left(\frac{C_{a}}{C_{i}}+1\right)-\delta_{\text{out}}^{18}\frac{C_{a}}{C_{i}}\right)] \end{array}$$

with $t^{18}=\frac{\left(1+a^{18}\right)\;E}{2\;g_{\mathrm{SC}}}$ and $a^{18}$ the fractionation for combined C^18^O^16^O diffusion across the stomata and boundary layer (Table S1). $\delta_{\mathrm{ce}}^{18}$ is the *δ* ^18^O of the CO_2_ in equilibrium with cytosol water (Cernusak *et al*., 2004), calculated as:

(Eqn 8) $$\delta_{\mathrm{ce}}^{18}=\delta_{w-e}^{18}\left(1+\varepsilon_{w}\right)+\varepsilon_{w}$$

$\varepsilon_{w}$ is the equilibrium ^18^O fractionation between chloroplast CO_2_ and water: $\varepsilon_{w}=\frac{17604}{T_{\text{leaf}}}-17.93$. $\delta_{w-e}^{18}$ is the *δ* ^18^O of the liquid water at evaporative sites in leaves, calculated as:

(Eqn 9) $$\delta_{w-e}^{18}=\delta_{w-E}^{18}+\varepsilon^{+}+\varepsilon_{K}+\left(\delta_{w-\text{out}}^{18}-\varepsilon_{K}-\delta_{w-E}^{18}\right)\frac{w_{\text{out}}}{\;w_{i}}$$

where $\delta_{w-\text{in}}^{18}$ and $\delta_{w-\text{out}}^{18}$ are the isotopic compositions of the H_2_O in the air entering and leaving the leaf cuvette; $\varepsilon^{+}$ is the equilibrium ^18^O fractionation between liquid water and vapour $\varepsilon^{+}=2.6-3.2\;\frac{1000}{T_{\text{leaf}}}+1.5\;\frac{10^{6}}{{T_{\text{leaf}}}^{2}}$, where *T*_leaf_ is the leaf temperature in Kelvin; $\varepsilon_{K}$ is kinetic fractionation during diffusion of H_2_^18^O from the substomatal air spaces to the atmosphere (Farquhar *et al*., 1989); $w_{\text{out}}$ and $w_{i}$ are the water vapour mole fractions in the air leaving the chamber and in the substomatal air spaces and $\delta_{w-E}^{18}$ is the *δ* ^18^O of water vapour transpired by the leaf, calculated as Craig *et al*. (1965):

(Eqn 10) $$\delta_{w-E}^{18}=\frac{w_{\text{out}}{\;\delta }_{w-\text{out}}^{18}-w_{\text{in}}{\;\delta }_{w-\text{in}}^{18}+\left(\delta_{w-\text{in}}^{18}-\delta_{w-\text{out}}^{18}\right)w_{\text{out}}\;w_{\text{in}}}{w_{\text{out}}-w_{\text{in}}}$$

where $w_{\text{in}}$ is the water vapour mole fractions in the air entering the leaf chamber.

### Unsaturation of the intercellular airspace

*C*_i_ is calculated from the stomatal conductance to CO_2_, which is, in turn, derived from the total conductance to water vapour (*g*_tw_; von Caemmerer & Farquhar, 1981):

(Eqn 11) $$g_{\mathrm{tw}}=\frac{E\;\left(1-\frac{1}{2}\left(w_{i}+w_{a}\right)\;\right)}{w_{i}-w_{a}}$$

To estimate the humidity of the intercellular airspaces, *w*_i_ in Eqns 9 and 11 was substituted by *h*_i_ *w*_s_, where *h*_i_ represents the relative humidity in the airspace, and *w*_s_ represents the saturation mole fraction at leaf temperature and air pressure (Cernusak *et al*., 2024). Initially, a dummy value for *h*_i_ was used to calculate both the *δ* ^18^O of the CO_2_ in equilibrium with cytosolic water ($\delta_{\mathrm{ce}}^{18}$; Eqn 8) and the *δ* ^18^O of CO_2_ at the sites of carbonic anhydrase activity ($\delta_{\mathrm{ca}}^{18}$):

(Eqn 12) $$\delta_{\mathrm{ca}}^{18}=\delta_{A}^{18}\left(1-\frac{C_{i}}{\;C_{\mathrm{ca}}}\right)\left(1+a_{m}^{18}\right)+\frac{C_{i}}{\;C_{\mathrm{ca}}}\;\left(\delta_{i}^{18}-a_{m}^{18}\right)+a_{m}^{18}$$

where *C*_ca_ is the CO_2_ mole fraction at the site of the isotopic equilibrium, which is defined as: Cca=Ci−AgMO. ^O^ *g*_M_ was derived under conditions in which the airspaces are assumed fully saturated (*h*_i_ = 100%) by calculating Eqn 6 for those datapoints with the lowest mole fraction difference (*w*_s_ − *w*_a_), following Cernusak *et al*. (2018) and Cernusak *et al*. (2019). Assuming the *δ* ^18^O of the CO_2_ in equilibrium with water matches that at the sites of carbonic anhydrase, *h*_i_ was solved for by minimising the squared difference between $\delta_{\mathrm{ce}}^{18}$ and $\delta_{\mathrm{ca}}^{18}$.

### Biochemical modelling

The ratio of Rubisco oxygenation relative to carboxylation was calculated as $\varphi =\frac{2\Gamma^{*}}{C_{M}}$, where $C_{M}=C_{i}-\frac{A}{g_{M}}$. At steady state, the actual rate of NADPH and ATP production equal their rate of consumption, we can therefore write $j_{\text{NADPH}}=\left(2+2\varphi \right)\;V_{C}$, and $j_{\mathrm{ATP}}=\left(3+3.5\varphi \right)\;V_{C}$ (eqns 6 and 7 in Farquhar *et al*. (1980), or eqns 2.5 and 2.6 in von Caemmerer (2000)). *V*_C_ is Rubisco's rate of carboxylation and can be described through a Michaelis–Menten equation $V_{C}=\frac{V_{\text{CMAX}}\;C_{M}}{C_{M}+k^{\prime }}$, where $k^{\prime }$ is Rubisco's Michaelis–Menten constant for CO_2_ adjusted for O_2_ inhibition, here 550 μmol mol^−1^. By combining, *V*_CMAX_ is solved as:

(Eqn 13) $$\begin{array}{l} V_{\text{CMAX}\left(j\right)}=\frac{j_{\text{NADPH}}\left(C_{M}+k^{\prime }\right)}{C_{M}\left(2+2\varphi \right)}\left(a\right),\text{or}\\ V_{\text{CMAX}\left(j_{\mathrm{ATP}}\right)}=\frac{j_{\mathrm{ATP}}\left(C_{M}+k^{\prime }\right)}{C_{M}\left(3+3.5\varphi \right)}\left(b\right) \end{array}$$

### Oxygen sensitivity

We quantified the sensitivity of the assimilation rate to oxygen as an elasticity, that is the fractional change in assimilation per fractional change in the oxygen mole fraction:

(Eqn 14) $$\eta {\left(A\right)}_{O_{2}}=\frac{A_{\mathrm{Low}}-A}{A}/\frac{O_{\mathrm{Low}}-O_{\mathrm{Amb}}}{O_{\mathrm{Amb}}}$$

where *A*_Low_ and *A* are assimilation rates measured under low and ambient oxygen, respectively. Elasticity of *Y*(II) to the oxygen mole fraction was calculated by substituting *A* for *Y*(II) in Eqn 14.

### Statistical analysis

Individual split‐line regressions of the relationships between *Ψ*_L_ and *A*, *g*_S_, *L*_S_ and *L*_NS_ were obtained by sequentially shifting the breakpoint to maximise the total residual sum of squares of the two regressions. A linear mixed‐effects model using the R (R Core Team, 2023) package lme4 (Bates, 2014) was used to analyse regression coefficients and the water potential breakpoints. Pairwise comparisons within species were made using the package emmeans with *P*‐values adjusted by the Tukey method and degrees of freedom corrected according to the Kenward–Roger method (Lenth, 2024).

Estimations of *g*_M_ are typically characterized by high variance, often requiring data filtering based on arbitrary criteria (Harley *et al*., 1992; Gilbert *et al*., 2012; Stangl *et al*., 2019; Knauer *et al*., 2022), risking introducing bias, or, when overconservative, discarding potentially valid information. To mitigate this, we fitted to the relationship between *Ψ*_L_ and *g*_M_ using a quantile generalised additive model (Fasiolo *et al*., 2021), which provides a robust fit that is less sensitive to outliers than ordinary least squares regressions. The model describes the median and the 95% confidence interval using a smoothing spline that captures nonlinear trends without assuming a specific functional form. The relationships between *Ψ*_L_ and *g*_M_, *j*_NADPH_, *V*_CMAX_, *η*(A)_O2_, or *η*(YII)_O2_ were evaluated using a Kendall's *τ*_b_ rank correlation (via the wdm package; Nagler, 2025), providing a nonparametric test that is independent of the normality or linearity assumptions inherent in regression models. Where possible, significance was evaluated via a stratified bootstrap approach to account for potential individual plant effects. To assess whether the observed nonstomatal limitation (*L*_NS_) was driven solely by *g*_M_, we modelled the hypothetical *g*_M_ required to fully account for the observed *L*_NS_ (Methods S4). We then compared modelled with measured *g*_M_ using a one‐sided Wilcoxon Signed‐Rank test. A higher measured than modelled *g*_M_ indicates that processes other than changes in *g*_M_ contribute to the observed *L*_NS_.

## Results

### Net assimilation of fully hydrated leaves

The responses of assimilation rates to stepwise changes in PPFD and CO_2_ mole fraction were typical for healthy C_3_ plants. As expected, under low [O_2_], both *A*/*C*_i_ and *A*/PPFD curves had a steeper initial slope, and higher levels of assimilation were observed under high PPFD (Fig. 1). Respiration in the light (*R*_Light_) and the parameters defining the shape of the *A*/*C*_i_ and *A*/*C*_M_ curves are shown in Table 1.

### Gas exchange during leaf dehydration

When plants were progressively pulled out of the solution, net assimilation (*A*) and CO_2_ mole fraction in the substomatal cavity (*C*_i_) gradually decreased (Fig. S3). At a leaf water potential (*Ψ*_L_) of *c*. −1 MPa, this decrease sharply accelerated. Stomatal conductance (*g*_S_) showed a similar decrease, but only after a small initial increase, confirming the well‐documented wrong‐way response (Buckley, 2019). Both stomatal and nonstomatal limitations (*L*_S_ and *L*_NS_) remained near zero until a threshold was reached – defined by a sharp change in *Ψ*_L_ – after which both limitations markedly increased (Fig. S3A–F). *L*_S_ was initially negligible for a broad interval of *Ψ*_L_ but eventually increased, five times more steeply (*P* = 0.0001) in wheat than sunflower (Fig. S3G,H). In both species, *L*_NS_ increased after a *Ψ*_L_ of *c*. −1.2 MPa; the increase was twice as steep in wheat as in sunflower (*P* = 0.02) and did not show a saturation maximum (Fig. S3I,J), consistent with the winter crop wheat being worse adapted to withstand such dehydration than the summer crop sunflower.

**Fig. 2:**
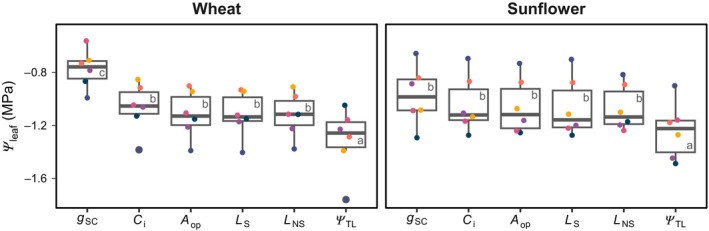
Incipient point of response and turgor loss. Leaf water potential (*Ψ*_L_) at the turgor loss point (*Ψ*_TL_), or at which a significant change in the slope (defined as the incipient point of response) of stomatal conductance (*g*_SC_), substomatal CO_2_ (*C*_i_), net assimilation rate (*A*_op_), stomatal limitation (*L*_S_), and nonstomatal limitation (*L*_NS_) was detected (Supporting Information Fig. S3). Horizontal lines within the boxes represent the median; circles indicate the incipient points for individual replicates. Different letters indicate a significant difference between the means estimated from a linear mixed effect model followed by a *post hoc* test (Tukey method).

The response to dehydration of each pair (*Ψ*_L_, *A*), (*Ψ*_L_, *C*_i_), (*Ψ*_L_, *g*_S_), (*Ψ*_L_, *L*_S_), and (*Ψ*_L_, *L*_NS_), was captured by individual split‐line regressions fitted to each replicate; the responses averaged over six replicates are shown as black lines in Fig. S3. The slope of the regression lines mathematically corresponds to sensitivity to dehydration, while the *Ψ*_L_ at the point in which the slope changed was defined as the point of incipient response (Fig. 2). The incipient point of response was compared with the turgor loss point measured on different leaves of the same plant batch (Fig. 2). In both species, incipient changes in gas exchange were *c*. 0.2 MPa above the turgor loss point (*P* < 0.05 in sunflower and *P* < 0.001 in wheat). The incipient point of stomatal closure was higher than that of all the other photosynthetic responses (Fig. 2), although the effect was statistically significant only in wheat (−0.77 MPa, *P* < 0.001). This aligns with previous results on drought‐stressed poplar clones, in which changes in leaf hydraulic conductance and *g*_S_ preceded the decrease in assimilation rates (Théroux‐Rancourt *et al*. 2014). With the exception of wheat *g*_S_, all quantities started to change at similar *Ψ*_L_ (−1.1 MPa), less negative than the turgor loss point (−1.2 MPa for sunflower or −1.3 MPa for wheat Fig. 2). The overall pattern agrees with results of Trueba *et al*. (2019) who compared 10 different Angiosperm species and concluded that stomatal closure generally precedes the turgor loss point. Our results also clearly show that stomatal closure does not prevent the rise of *L*_NS_, supporting the optimal control model of Dewar *et al*. (2022), which posits that stomata may adjust to, rather than induce, nonstomatal limitation.

### Mesophyll conductance with three methods

**Fig. 3:**
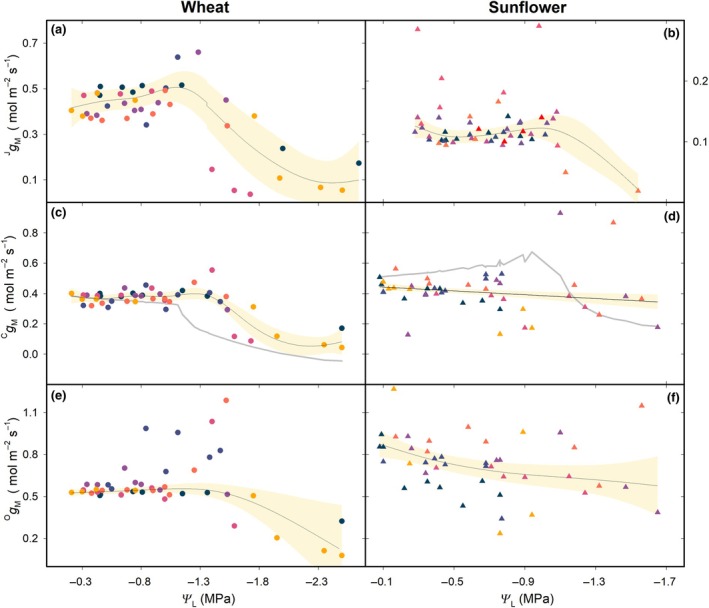
Mesophyll conductance under dehydration. Relationship between the leaf water potential (*Ψ*_L_) and mesophyll conductance derived from Chl fluorescence measurements a,b) (^J^ *g*_M_), stable carbon isotope discrimination c,d) (^C^ *g*_M_), and stable oxygen isotope discrimination e,f) (^O^ *g*_M_). The solid line represents the median trend of the data estimated via a quantile generalized additive model. The yellow ribbon indicates the approximate 95% confidence interval (±1.96 times the SE) around the fit. Grey lines represent the calculated ^C^ *g*_M_ assuming the observed decrease in nonstomatal limitation (*L*_NS_) was only due to ^C^ *g*_M_.

For each datapoint measured under dehydration (Fig. S4), we derived *g*_M_ from the total rate of electron transport (^*J*^ *g*_M_), from carbon isotope discrimination (^C^ *g*_M_), and from oxygen isotope discrimination (^O^ *g*_M_). In sunflower, ^*J*^ *g*_M_ was significantly lower than ^C^ *g*_M_ and ^O^ *g*_M_. This is likely due to the fact that in sunflower, ^*J*^ *g*_M_ was obtained under a higher reference CO_2_ mole fraction (800 μmol mol^−1^), while ^C^ *g*_M_ and ^O^ *g*_M_ were obtained under 300 μmol mol^−1^ (Fig. 3b,d,f). A lower *g*_M_ under high CO_2_ levels has consistently been observed (Flexas *et al*., 2007; Vrábl *et al*., 2009; Schaeufele *et al*., 2011; Mizokami *et al*., 2015, 2019), although the mechanisms underlying such effect are unclear. Our ^C^ *g*_M_ values align with previous reports both in sunflower (0.45 mol m^−2^ s^−1^ in Schaeufele *et al*. (2011); 0.41 mol m^−2^ s^−1^ in Vrábl *et al*. (2009)) and in wheat (*c*. 0.4 mol m^−2^ s^−1^ in Tazoe *et al*. (2009) and *c*. 0.5 mol m^−2^ s^−1^ in Loreto *et al*. (1994)). ^O^ *g*_M_ was higher than ^C^ *g*_M_ in both wheat and sunflower (Fig. 3) in agreement with previous reports (Gillon & Yakir, 2000; Ogée *et al*., 2018; Holloway‐Phillips *et al*., 2019) (Fig. 3a,c,e). Although the exact boundary between ^C^ *g*_M_ and ^O^ *g*_M_ is unclear, ^C^ *g*_M_ represents conductance to Rubisco sites – a longer path than ^O^ *g*_M_, which likely reflects conductance to the sites of isotopic equilibrium between leaf water and CO_2_ (Ogée *et al*., 2018). Isoforms of carbonic anhydrases – fast enzymes catalysing the hydration of CO_2_ – are located in the chloroplast, cytosol, plasma membrane (DiMario *et al*., 2017), and the cell wall (Weerasooriya *et al*., 2024). Our results support the view that dehydration impairs the entire diffusion path, including external elements, as reviewed by Flexas *et al*. (2021).

Mesophyll conductance decreased with dehydration (Kendall's *τ*_b_ rank correlation; sunflower *P* = 0.61, 0.037 and 0.0016 for ^*J*^ *g*_M_, ^C^ *g*_M_, and ^O^ *g*_M_; wheat *P* = 0.046, 0.042 and 0.091). The relationship between *g*_M_ and *Ψ*_L_ exhibited a threshold‐like response, especially in wheat in which the trend line reveals a distinct change in slope *c*. −1.5 MPa, marking a transition to a more rapid decrease in *g*_M_ at lower *Ψ*_L_ (Fig. 3). Regression of *g*_M_ against assimilation is in Fig. S5.

### Biochemical modelling

**Fig. 4:**
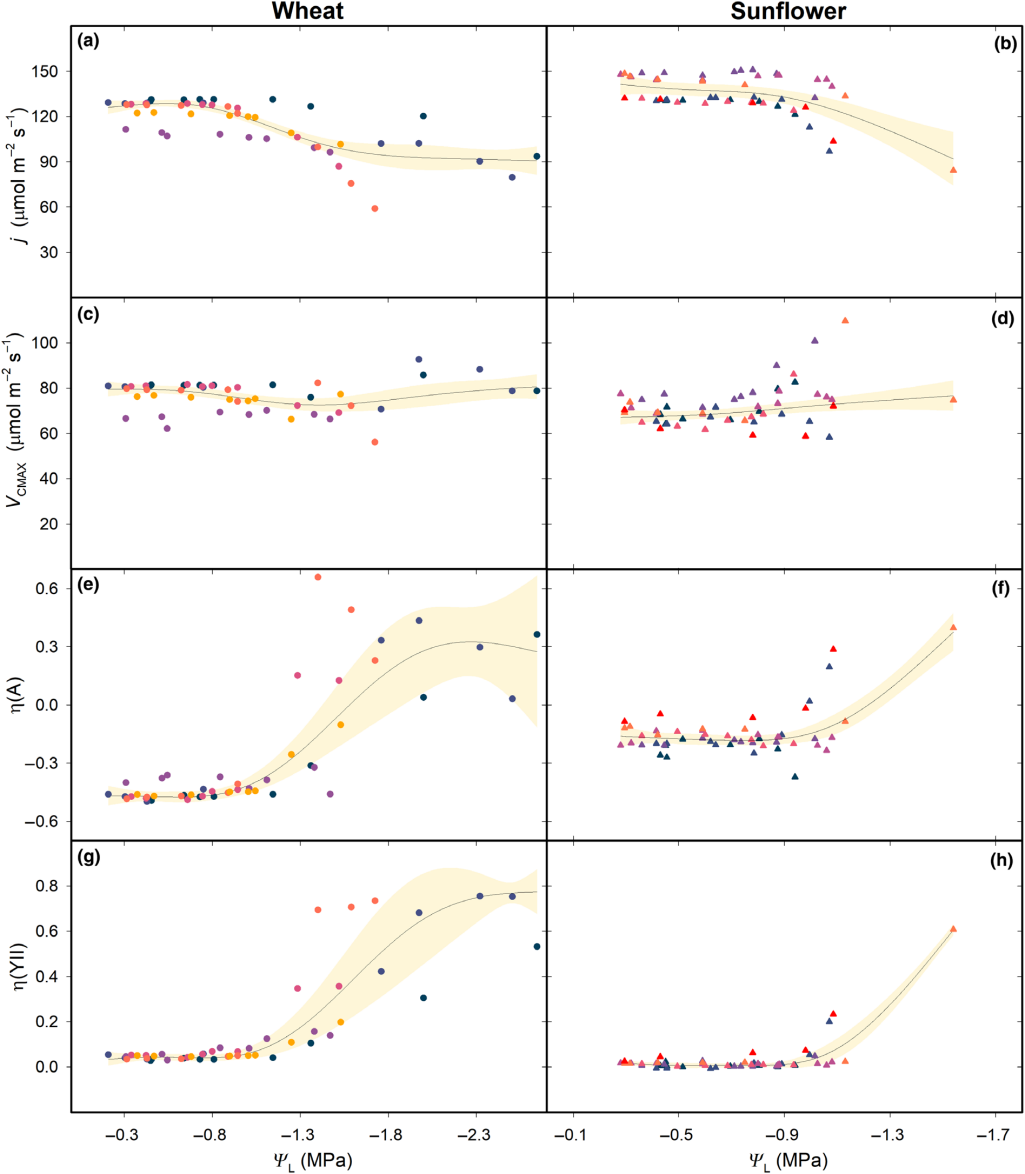
Electron transport, carboxylation capacity, and the oxygen relative sensitivity of the net assimilation rate and the photosystem II yield. Relationship between the leaf water potential (*Ψ*_L_) and electron transport rates estimated from Chl fluorescence (*j*, (a, b) results obtained from *j*_ATP_ through Eqn 13b were identical, available in Supporting Information Dataset S1), Rubisco carboxylation capacity (V_CMAX_ obtained from Eqn 13a, panels (c and d; values obtained from Eqn 13b were similar and are not shown), the oxygen relative sensitivity (elasticity) of the net assimilation rate (*η*(*A*) (e, f)), and the oxygen elasticity of the operating efficiency of photosystem II (*η*(*Y*II) (g, h)). *n* = 6, biological replicates are shown in different colours.

Electron transport rates (*j*) and Rubisco carboxylation capacity (*V*_CMAX_) were estimated from the gas‐exchange and Chl‐fluorescence data. Fig. 4 shows that under both low and ambient O_2_ conditions, *V*_CMAX_ estimates stayed essentially constant (Kendall *τ*_b_ rank correlation *P* > 0.05; results of Eqn 13b did not differ), but *j* decreased with dehydration even when accounting for individual‐level variability through bootstrapping (wheat: *τ*_b_ = 0.52, *P* < 0.001, sunflower: *τ*_b_ = 0.27, *P* = 0.025).

## Discussion

Estimating *g*_M_ requires precise ^13^CO_2_ and accurate substomatal CO_2_ mole fractions (*C*_i_). In C_3_ plants stomatal conductance and discrimination against ^13^CO_2_ inherently high, but uncertainty increases with dehydration as stomatal closure decreases *C*_i_/*C*_a_ (e.g. Eqn 5), presenting technical challenges and trade‐offs (see Methods S2; Note S2). Heterogeneity in stomatal closure (patchiness) could lead to an apparent change in *g*_M_ (Laisk, 1983; Terashima *et al*., 1988; Mott & Buckley, 2000; Rockwell *et al*., 2022). While heterogeneous stomatal closure cannot be entirely excluded, our experimental conditions and the absence of characteristic indicators of patchiness suggest that its contribution to the observed results was minimal (Note S3).

**Fig. 5:**
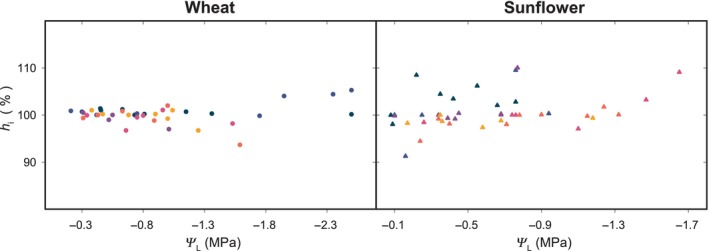
Relative humidity of the intercellular airspace, estimated by ^18^O discrimination. Relationship between the leaf water potential (*Ψ*_L_) and the estimated relative humidity in the intercellular airspace (*h*_i_) for wheat and sunflower. *n* = 6, biological replicates are shown in different colours.

*C*_i_ can also be affected by the typical assumption of 100% substomatal airspace relative humidity (*h*_i_). We estimated *h*_i_ using the stable oxygen isotope method (Cernusak *et al*., 2018) and found no evidence for *h*_i_ decreasing below 90% (Fig. 5). While smaller differences could not be resolved, a sensitivity analysis indicated that assuming a lower *h*_i_ only marginally increased the relative difference in *g*_M_ between hydrated and dehydrated states (Fig. S6). This suggests that shifts in *h*_i_ are insufficient to explain the observed *g*_M_ patterns, reinforcing that the decrease is driven by broader physiological or hydraulic constraints. The sensitivity analysis further showed that underestimating leaf temperature would reduce the observed decrease in *g*_M_ under dehydration, but substantial error seems unlikely (Note S1). The impact of varying cuticular conductance (Fig. S6) and the typical assumption that CO_2_ and water reach isotopic equilibrium in the cytosol (Note S4) would also not appreciably affect the difference in *g*_M_. We therefore deem these assumptions broadly valid and appropriate for this study.

The rise in nonstomatal limitation under dehydration was linked to a decrease in *g*_M_ (Fig. 3). A similar decrease in *g*_M_ was found in sunflower and tobacco leaves subject to fast dehydration upon severing the petiole (Flexas *et al*., 2006a), although a mechanism for this response was not identified in that study. Wilting is known to induce anatomical changes, which may affect *g*_M_. Dehydration can shrink mesophyll cells (Canny & Huang, 2006; Canny *et al*., 2011), which may explain the thinner leaves with reduced airspace volume observed by Scoffoni *et al*. (2014). However, only quite drastic porosity changes are thought to affect *g*_M_ (Lehmeier *et al*., 2017). Scoffoni *et al*. (2014) observed that anatomical changes can precede the wilting stage. This may include changes in cell shape and size that reduce mesophyll or chloroplast surface area exposed to intercellular spaces, potentially impinging on *g*_M_ (Evans *et al*., 1994; Clarke *et al*., 2021). Besides these anatomical effects, biochemical factors influencing diffusion can also be affected by dehydration. A decrease in the activity of aquaporins (Miyazawa *et al*., 2008) or in the expression levels of aquaporin and carbonic anhydrase genes (Perez‐Martin *et al*., 2014) has been observed under drought conditions. Aquaporins are thought to, possibly in combination with carbonic anhydrases, speedup CO_2_ diffusion into cells (Mizokami *et al*., 2022) potentially affecting *g*_M_ (Flexas *et al*., 2018). Interestingly, aquaporins are also a key factor in controlling the transport and distribution of water in leaves (Sade *et al*., 2014; Secchi & Zwieniecki, 2014), and a decrease in abundance or activity may therefore also reduce *g*_M_ indirectly by altering the size and shape of mesophyll cells, see for example Hanba *et al*. (2004). Mizokami *et al*. (2015) and Mizokami *et al*. (2019) showed that exogenous abscisic acid can rapidly decrease *g*_M_ in a dose‐dependent manner, which could result from rapid aquaporin gating via phosphorylation. Internalisation (the sequestration of membrane proteins into cytoplasmic vesicles) has also been suggested to reduce aquaporin plasma membrane abundance under dehydration (Mizokami *et al*., 2022). Nevertheless, the role of aquaporins in CO_2_ diffusion remains controversial (Kromdijk *et al*., 2020; Clarke *et al*., 2022).

The co‐occurrence of a decrease in *g*_M_ with an increase in *L*_NS_ does not demonstrate that *g*_M_ is causing *L*_NS_. To investigate the maximum extent to which *g*_M_ could cause *L*_NS_, we calculated the *g*_M_ values that would be observed if *g*_M_ alone was responsible for *L*_NS_ (Methods S4). If *g*_M_ were the sole cause of the observed rise of *L*_NS_, the predicted *g*_M_ (grey plots in Fig. 3c,d) would describe the trend of the measured data (black lines). A Wilcoxon Signed‐Rank test showed that observed *g*_M_ was significantly higher (sunflower *P* = 0.049, wheat *P* = 0.020) than the values required to explain *L*_NS_, suggesting that factors beyond mesophyll conductance contribute to the observed nonstomatal limitation. We observed a decreased electron transport rate (*j*, Fig. 4a,b) but Rubisco *V*_CMAX_ was not affected (Fig. 4c,d). This agrees with earlier results showing that under short‐term water stress, RuBP regeneration capacity decreased, but *V*_CMAX_ and *in vitro* Rubisco activity were not much affected (Sharkey & Badger, 1982; Von Caemmerer & Farquhar, 1984). A link between *g*_M_ and light reaction capacity was observed in tobacco lines overexpressing or antisense of the aquaporin gene NtAQP1, which had higher and lower *g*_M_ than the wild‐type, respectively, with parallel changes in *j* but no differences in *V*_CMAX_ (Flexas *et al*., 2006b), mirroring our results. *j* is attuned to demand from carbon reactions (Roach & Krieger‐Liszkay, 2014). In essence, lumen acidification slows plastoquinol oxidation by cytochrome *b*_6_ *f* – photosynthetic control (Tikhonov, 2014), induces nonphotochemical energy dissipation in PSII antennae (Li *et al*., 2004), and slows PSI by keeping the donor side oxidised (Takagi *et al*., 2016; Ermakova *et al*., 2024).

Our data support light reaction downregulation, as indicated by the fivefold increase in *Y*(II) elasticity to [O_2_] over the course of dehydration in wheat (*τ*_b_ = −0.63, *P* < 0.001; Fig. 4g). We previously proposed for maize and sorghum exposed to rapid dehydration that a reduction in ATP and NADPH demand would be caused by a Rubisco slowdown (Bellasio *et al*., 2024). Rubisco activity is controlled by Rubisco activase, phosphoglycerate feedback inhibition, and light modulation (Portis Jr, 1992). Rubisco can also be degraded, but this is typically only observed under severe and prolonged drought, in which high‐light intensities and low chloroplast CO_2_ resulting from reduced and *g*_M_ induce oxidative damage (Flexas *et al*., 2002, 2006c; Medrano *et al*., 2002; Zhou *et al*., 2007). For the C_3_ plants examined here, we found *V*_CMAX_ invariant (wheat: *τ*_b_ = 0.11, *P* = 0.40, sunflower: *τ*_b_ = −0.18, *P* = 0.17; Fig. 4c,d); therefore, Rubisco downregulation or degradation is unlikely. However, light reaction downregulation can also be a result of phosphate limitation (Furbank *et al*., 1987): a decrease in phosphate concentration reduces ATP synthase turnover due to substrate limitation, which traps protons in the lumen. This acidification then acts as a brake on electron transport triggering nonphotochemical energy dissipation. In support of this notion, we observed a reversal of assimilation‐elasticity to low oxygen, which became positive when *Ψ*_L_ fell below −1.5 MPa in wheat (*τ*_b_ = 0.60, *P* < 0.001; Fig. 4e). This condition is indicative of phosphate limitation (Sharkey, 1985). The link between oxygen sensitivity and phosphate limitation is well‐supported by both experimental (Sharkey, 1985; Stitt, 1986) and theoretical evidence (Harley & Sharkey, 1991; Bellasio, 2019). Nonetheless, the evidence remains indirect, and direct quantification of phosphoglycerate, phosphate, and sugar phosphates would be advisable in future studies.

**Fig. 6:**
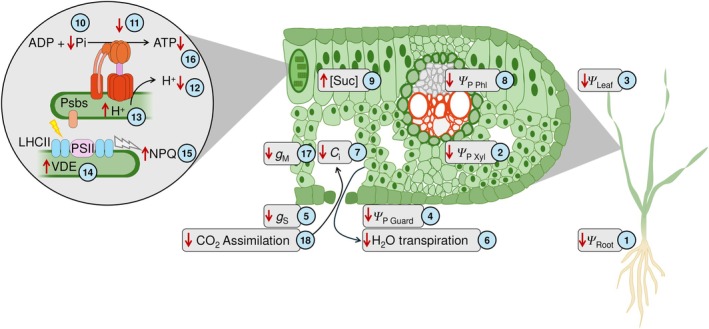
Schematic representation of the causal sequence through which rapid water loss imposes diffusive and nondiffusive restrictions on carbon assimilation. Dehydration (1) reduces xylem pressure (2) and leaf water potential (3), which lowers guard cell turgor (4), stomatal conductance (5), transpiration (6), and intercellular CO_2_ mole fraction, *C*_i_ (7), leading to stomatal limitation. In parallel, phloem turgor (8) decreases, slowing phloem loading and sugar export and causing sucrose accumulation in the mesophyll (9). This is followed by triose phosphate accumulation, which slows phosphoglycerate reduction and leads to its buildup. This feedback inhibits Rubisco activity in the chloroplast, resulting in nonstomatal limitation. At the same time, inorganic phosphate becomes sequestered in sugar phosphates and phosphoglycerate, reducing substrate availability (10) and thus slowing ATP synthase activity (11). This has two consequences: first, proton efflux from the lumen is reduced (12), causing lumen acidification (13) and activation of Psbs and violaxanthin de‐epoxidase, VDE (14), leading to nonphotochemical energy dissipation (15) in the photosystem II antennae (LHCII); second, ATP production slows. Reduced ATP concentration (16) further limits Rubisco activity (a second nonstomatal limitation component) and promotes dephosphorylation of CO_2_ porins at the mesophyll plasma membrane, lowering mesophyll conductance (17) – a third nonstomatal limitation. Together, these processes result in a drop of CO_2_ assimilation (18).

Phosphate limitation can occur when leaves are exposed to oversaturating CO_2_, causing Rubisco carboxylation to outpace reduction, trapping phosphate in phosphoglycerate (Bellasio, 2019), slowing ATP synthesis. The high phosphoglycerate‐to‐phosphate ratio activates sucrose and starch synthesis, which release phosphate (Stitt & Quick, 1989), recovering photophosphorylation – at least in part (Busch *et al*., 2018) – sometimes with fascinating oscillations that dampen over time (Laisk & Eichelmann, 1989; Walker, 1992). However, this scenario is unlikely here, as for most of our data, Rubisco was substrate‐limited by subambient CO_2_ levels (*c*. 350 μmol mol^−1^), and moderate PPFD (between 500 and 900 μmol m^−2^ s^−1^) that restrained RuBP supply. Instead, we propose that a phosphate shortage would be due to product‐inhibition of phosphate‐releasing reactions (Fig. 6). Under mild water stress, both starch and sucrose synthesis are strongly inhibited (Huber, 1989; Sharkey & Seemann, 1989; Vassey & Sharkey, 1989). The mechanism behind the downregulation of starch synthesis remains unclear, but sucrose synthesis could be inhibited by sucrose accumulation in leaves, resulting from decreased sugar export capacity (Paul & Foyer, 2001). This likely occurs under rapid dehydration, in which a drop in *Ψ*_L_ reduces phloem turgor pressure (Smith & Milburn, 1980), hindering sap flow and sucrose transport to sink organs (Sevanto, 2018) and leading to sucrose accumulation in the mesophyll.

Several phenomena associated with phosphate limitation are agronomically relevant. Midday depression of photosynthesis is widespread in crops, in which transient reductions in water potential driven by peak irradiance and evaporative demand can constrain daily carbon gain more than chronic drought (Xu & Shen, 1996). In addition, CO_2_ fertilisation often fails to produce the expected productivity gains in both C_3_ and C_4_ plants (Morgan *et al*., 2005; Leakey *et al*., 2006), a response that has been partly attributed to phosphate availability (Ellsworth *et al*., 2017). The oxygen sensitivity of assimilation and *Y*(II) proposed here provides a practical diagnostic of phosphate limitation, allowing real‐time assessment of metabolic constraints on plant performance. Importantly, *η*(*A*)O_2_ and *η*(*Y*II)O_2_ can be derived from data obtained using an established rapid protocol based on low‐oxygen transitions in photosynthesising leaves (Bellasio *et al*. 2014), without modification of the measurement routine.

### Conclusion

Assimilation dropped before loss of turgor due to a combination of stomatal and nonstomatal factors. Part of the nonstomatal limitation was attributable to restricted CO_2_ diffusion to the chloroplasts, driven by reduced mesophyll conductance. In parallel, a biochemical limitation emerged, more likely due to a slowdown in ATP synthesis driven by phosphate limitation than to a reduction in Rubisco carboxylation capacity.

## Competing interests

None declared.

## Author contributions

CB conceived the project, acquired funding and designed the experiment with contributions of JF. HS‐W provided isotopic methods. GDF provided laboratories and resources. CB performed the research with contributions of HS‐W. CB coded and ran the models. DT and CB analysed the data. CB, DT, JF, HS‐W and GDF wrote the paper. CB and DT contributed equally to this work.

## Disclaimer

The New Phytologist Foundation remains neutral with regard to jurisdictional claims in maps and in any institutional affiliations.

## Supporting information


**Dataset S1** Sunflower and wheat gas exchange and fluorescence under rapid dehydration.

**Fig. S1** Example of pressure–volume curve obtained for wheat.
**Fig. S2** Experimental Set‐up.
**Fig. S3** Relationship between gas‐exchange variables and leaf water potential.
**Fig. S4** Fluorescence and isotope discrimination variables in relation to water potential.
**Fig. S5** The relationship between net assimilation and mesophyll conductance estimated by three different methods.
**Fig. S6** Sensitivity analysis of the effect of cuticular conductance, unsaturation and leaf temperature on the differences in mesophyll conductance between hydrated and dehydrated leaves.
**Methods S1** Hydromechanical characterisation.
**Methods S2** Accounting for cuticular conductance and unsaturation in the calculation of gas‐exchange variables.
**Methods S3** Modelling gas exchange on hydrated plants.
**Methods S4** Modelling mesophyll conductance from given values of limitations.
**Notes S1** Uncertainties in stomatal transpiration, airspace unsaturation, and leaf temperature.
**Notes S2** *Ψ*_L_ heterogeneity.
**Notes S3** Stomatal patchiness.
**Notes S4** Oxygen Isotopic Equilibrium.
**Table S1** Abbreviations, definitions and units for variables and acronyms described in the text.
Please note: Wiley is not responsible for the content or functionality of any Supporting Information supplied by the authors. Any queries (other than missing material) should be directed to the *New Phytologist* Central Office.

## Data Availability

Data are available in the Supporting Information (Dataset S1).

## References

[nph71236-bib-0001] Angus J , Moncur M . 1977. Water stress and phenology in wheat. Australian Journal of Agricultural Research 28: 177–181.

[nph71236-bib-0002] Barbour MM , Evans JR , Simonin KA , von Caemmerer S . 2016. Online CO_2_ and H_2_O oxygen isotope fractionation allows estimation of mesophyll conductance in C_4_ plants, and reveals that mesophyll conductance decreases as leaves age in both C_4_ and C_3_ plants. New Phytologist 210: 875–889. 26778088 10.1111/nph.13830

[nph71236-bib-0003] Bates D . 2014. Fitting linear mixed‐effects models using lme4. *arXiv*: 1406.5823.

[nph71236-bib-0004] Bellasio C . 2019. A generalised dynamic model of leaf‐level C_3_ photosynthesis combining light and dark reactions with stomatal behaviour. Photosynthesis Research 141: 99–118. 30471008 10.1007/s11120-018-0601-1

[nph71236-bib-0005] Bellasio C . 2025. Quantifying photosynthetic restrictions. Photosynthesis Research 163: 19. 39964589 10.1007/s11120-024-01129-y PMC11835928

[nph71236-bib-0006] Bellasio C , Beerling DJ , Griffiths H . 2016. An Excel tool for deriving key photosynthetic parameters from combined gas exchange and chlorophyll fluorescence: theory and practice. Plant, Cell & Environment 39: 1180–1197. 10.1111/pce.12560 25923517

[nph71236-bib-0007] Bellasio C , Burgess SJ , Griffiths H , Hibberd JM . 2014. A high throughput gas exchange screen for determining rates of photorespiration or regulation of C_4_ activity. Journal of Experimental Botany 65: 3769–3779. 25006037 10.1093/jxb/eru238 PMC4085971

[nph71236-bib-0008] Bellasio C , Farquhar GD . 2019. A leaf‐level biochemical model simulating the introduction of C_2_ and C_4_ photosynthesis in C_3_ rice: gains, losses and metabolite fluxes. New Phytologist 223: 150–166. 30859576 10.1111/nph.15787

[nph71236-bib-0009] Bellasio C , Griffiths H . 2014. Acclimation of C_4_ metabolism to low light in mature maize leaves could limit energetic losses during progressive shading in a crop canopy. Journal of Experimental Botany 65: 3725–3736. 24591058 10.1093/jxb/eru052 PMC4085954

[nph71236-bib-0010] Bellasio C , Stuart‐Williams H , Farquhar GD , Flexas J . 2023. C_4_ maize and sorghum are more sensitive to rapid dehydration than C_3_ wheat and sunflower. New Phytologist 240: 2239–2252. 37814525 10.1111/nph.19299

[nph71236-bib-0011] Bellasio C , Stuart‐Williams H , Farquhar GD , Flexas J . 2024. Fast dehydration reduces bundle sheath conductance in C_4_ maize and sorghum. New Phytologist 244: 2197–2209. 39460370 10.1111/nph.20167 PMC11579431

[nph71236-bib-0012] Biscoe P , Cohen Y , Wallace J . 1976. Daily and seasonal changes of water potential in cereals. Philosophical Transactions of the Royal Society of London. B, Biological Sciences 273: 565–580.

[nph71236-bib-0013] Boyer J , Ghorashy S . 1971. Rapid field measurement of leaf water potential in soybean 1. Agronomy Journal 63: 344–345.

[nph71236-bib-0014] Boyer J , Johnson R , Saupe S . 1980. Afternoon water deficits and grain yields in old and new soybean cultivars 1. Agronomy Journal 72: 981–986.

[nph71236-bib-0015] Brand WA , Coplen TB , Vogl J , Rosner M , Prohaska T . 2014. Assessment of international reference materials for isotope‐ratio analysis (IUPAC technical report). Pure and Applied Chemistry 86: 425–467.

[nph71236-bib-0016] Buckley TN . 2019. How do stomata respond to water status? New Phytologist 224: 21–36. 31069803 10.1111/nph.15899

[nph71236-bib-0017] Busch FA , Holloway‐Phillips M , Stuart‐Williams H , Farquhar GD . 2020. Revisiting carbon isotope discrimination in C_3_ plants shows respiration rules when photosynthesis is low. Nature Plants 6: 245–258. 32170287 10.1038/s41477-020-0606-6

[nph71236-bib-0018] Busch FA , Sage RF , Farquhar GD . 2018. Plants increase CO_2_ uptake by assimilating nitrogen via the photorespiratory pathway. Nature Plants 4: 46–54. 29229957 10.1038/s41477-017-0065-x

[nph71236-bib-0019] von Caemmerer S . 2000. Biochemical models of leaf photosynthesis. Collingwood, Australia: CSIRO.

[nph71236-bib-0020] von Caemmerer S , Evans JR . 1991. Determination of the average partial‐pressure of CO_2_ in chloroplasts from leaves of several C_3_ plants. Australian Journal of Plant Physiology 18: 287–305.

[nph71236-bib-0021] von Caemmerer S , Farquhar GD . 1981. Some relationships between the biochemistry of photosynthesis and the gas exchange of leaves. Planta 153: 376–387. 24276943 10.1007/BF00384257

[nph71236-bib-0119] von Caemmerer S , Farquhar G . 1984. Effects of partial defoliation, changes of irradiance during growth, short‐term water stress and growth at enhanced p (CO_2_) on the photosynthetic capacity of leaves of *Phaseolus vulgaris* L. Planta 160: 320–329. 24258581 10.1007/BF00393413

[nph71236-bib-0022] Canny M , Huang C . 2006. Leaf water content and palisade cell size. New Phytologist 170: 75–85. 16539605 10.1111/j.1469-8137.2005.01633.x

[nph71236-bib-0023] Canny M , Wong SC , Huang C , Miller C . 2011. Differential shrinkage of mesophyll cells in transpiring cotton leaves: implications for static and dynamic pools of water, and for water transport pathways. Functional Plant Biology 39: 91–102. 10.1071/FP11172 32480764

[nph71236-bib-0024] Cernusak LA , Farquhar GD , Wong SC , Stuart‐Williams H . 2004. Measurement and interpretation of the oxygen isotope composition of carbon dioxide respired by leaves in the dark. Plant Physiology 136: 3350–3363. 15377777 10.1104/pp.104.040758 PMC523536

[nph71236-bib-0025] Cernusak LA , Goldsmith GR , Arend M , Siegwolf RTW . 2019. Effect of vapor pressure deficit on gas exchange in wild‐type and abscisic acid – insensitive plants1. Plant Physiology 181: 1573–1586. 31562233 10.1104/pp.19.00436 PMC6878010

[nph71236-bib-0026] Cernusak LA , Ubierna N , Jenkins MW , Garrity SR , Rahn T , Powers HH , Hanson DT , Sevanto S , Wong SC , McDowell NG . 2018. Unsaturation of vapour pressure inside leaves of two conifer species. Scientific Reports 8: 1–7. 29769592 10.1038/s41598-018-25838-2 PMC5955884

[nph71236-bib-0027] Cernusak LA , Wong SC , Stuart‐Williams H , Márquez DA , Pontarin N , Farquhar GD . 2024. Unsaturation in the air spaces of leaves and its implications. Plant, Cell & Environment 47: 3685–3698. 10.1111/pce.15001 38867619

[nph71236-bib-0028] Clarke VC , Danila FR , von Caemmerer S . 2021. CO_2_ diffusion in tobacco: a link between mesophyll conductance and leaf anatomy. Interface Focus 11: 20200040. 33628426 10.1098/rsfs.2020.0040 PMC7898150

[nph71236-bib-0029] Clarke VC , De Rosa A , Massey B , George AM , Evans JR , von Caemmerer S , Groszmann M . 2022. Mesophyll conductance is unaffected by expression of Arabidopsis *PIP1* aquaporins in the plasmalemma of *Nicotiana* . Journal of Experimental Botany 73: 3625–3636. 35184158 10.1093/jxb/erac065 PMC9162178

[nph71236-bib-0030] Cornic G , Prioul JL , Louason G . 1983. Stomatal and non‐stomatal contribution in the decline in leaf net CO_2_ uptake during rapid water stress. Physiologia Plantarum 58: 295–301.

[nph71236-bib-0031] Craig H , Gordon LI , Tongiorgi E . 1965. Stable isotopes in oceanographic studies and paleotemperatures. Pisa, Italy: V. Lischi e Figli, 122.

[nph71236-bib-0032] Dewar R , Hölttä T , Salmon Y . 2022. Exploring optimal stomatal control under alternative hypotheses for the regulation of plant sources and sinks. New Phytologist 233: 639–654. 34637543 10.1111/nph.17795

[nph71236-bib-0033] DiMario RJ , Clayton H , Mukherjee A , Ludwig M , Moroney JV . 2017. Plant carbonic anhydrases: structures, locations, evolution, and physiological roles. Molecular Plant 10: 30–46. 27646307 10.1016/j.molp.2016.09.001 PMC5226100

[nph71236-bib-0034] Dutt S , Gill K . 1978. Diurnal changes in leaf water potential of rice, barley and wheat. Biologia Plantarum 20: 472–474.

[nph71236-bib-0035] Ellsworth DS , Anderson Ian C , Crous Kristine Y , Cooke J , Drake John E , Gherlenda Andrew N , Gimeno Teresa E , Macdonald Catriona A , Medlyn Belinda E , Powell Jeff R *et al*. 2017. Elevated CO_2_ does not increase eucalypt forest productivity on a low‐phosphorus soil. Nature Climate Change 7: 279–282.

[nph71236-bib-0036] Ermakova M , Fitzpatrick D , Larkum AWD . 2024. Cyclic electron flow and Photosystem II‐less photosynthesis. Functional Plant Biology 51: FP24185. 39471160 10.1071/FP24185

[nph71236-bib-0037] Evans JR , Sharkey TD , Berry JA , Farquhar GD . 1986. Carbon isotope discrimination measured concurrently with gas exchange to investigate CO_2_ diffusion in leaves of higher‐plants. Australian Journal of Plant Physiology 13: 281–292.

[nph71236-bib-0038] Evans JR , von Caemmerer S , Setchell BA , Hudson GS . 1994. The relationship between CO_2_ transfer conductance and leaf anatomy in transgenic tobacco with a reduced content of Rubisco. Australian Journal of Plant Physiology 21: 475–495.

[nph71236-bib-0039] Farquhar G , Hubick K , Condon A , Richards R , Rundel P , Ehleringer J , Nagy K . 1989. Stable isotopes in ecological research. Ecological Studies 68: 21–40.

[nph71236-bib-0040] Farquhar GD , Cernusak LA . 2012. Ternary effects on the gas exchange of isotopologues of carbon dioxide. Plant, Cell & Environment 35: 1221–1231. 10.1111/j.1365-3040.2012.02484.x 22292425

[nph71236-bib-0041] Farquhar GD , Lloyd J . 1993. Carbon and oxygen isotope effects in the exchange of carbon dioxide between terrestrial plants and the athmosphere. In: Ehleringer JR , ed. Stable isotopes and plant carbon–water relations. New York, NY, USA: Academic Press, 47–70.

[nph71236-bib-0042] Farquhar GD , von Caemmerer S , Berry JA . 1980. A biochemical‐model of photosynthetic CO_2_ assimilation in leaves of C_3_ species. Planta 149: 78–90. 24306196 10.1007/BF00386231

[nph71236-bib-0043] Fasiolo M , Wood SN , Zaffran M , Nedellec R , Goude Y . 2021. qgam: bayesian nonparametric quantile regression modeling in R. Journal of Statistical Software 100: 1–31.

[nph71236-bib-0044] Flexas J , Bota J , Escalona JM , Sampol B , Medrano H . 2002. Effects of drought on photosynthesis in grapevines under field conditions: an evaluation of stomatal and mesophyll limitations. Functional Plant Biology 29: 461–471. 32689491 10.1071/PP01119

[nph71236-bib-0045] Flexas J , Bota J , Galmes J , Medrano H , Ribas‐Carbó M . 2006a. Keeping a positive carbon balance under adverse conditions: responses of photosynthesis and respiration to water stress. Physiologia Plantarum 127: 343–352.

[nph71236-bib-0046] Flexas J , Bota J , Loreto F , Cornic G , Sharkey T . 2004. Diffusive and metabolic limitations to photosynthesis under drought and salinity in C_3_ plants. Plant Biology 6: 269–279. 15143435 10.1055/s-2004-820867

[nph71236-bib-0047] Flexas J , Cano FJ , Carriquí M , Coopman RE , Mizokami Y , Tholen D , Xiong D . 2018. CO_2_ diffusion inside photosynthetic organs. In: The leaf: a platform for performing photosynthesis. Cham, Switzerland: Springer International, 163–208.

[nph71236-bib-0048] Flexas J , Clemente‐Moreno MJ , Bota J , Brodribb TJ , Gago J , Mizokami Y , Nadal M , Perera‐Castro AV , Roig‐Oliver M , Sugiura D . 2021. Cell wall thickness and composition are involved in photosynthetic limitation. Journal of Experimental Botany 72: 3971–3986. 33780533 10.1093/jxb/erab144

[nph71236-bib-0049] Flexas J , Diaz‐Espejo A , Galmes J , Kaldenhoff R , Medrano H , Ribas‐Carbo M . 2007. Rapid variations of mesophyll conductance in response to changes in CO_2_ concentration around leaves. Plant, Cell & Environment 30: 1284–1298. 10.1111/j.1365-3040.2007.01700.x 17727418

[nph71236-bib-0050] Flexas J , Ribas‐Carbó M , Bota J , Galmés J , Henkle M , Martínez‐Cañellas S , Medrano H . 2006c. Decreased Rubisco activity during water stress is not induced by decreased relative water content but related to conditions of low stomatal conductance and chloroplast CO_2_ concentration. New Phytologist 172: 73–82. 16945090 10.1111/j.1469-8137.2006.01794.x

[nph71236-bib-0051] Flexas J , Ribas‐Carbó M , Hanson DT , Bota J , Otto B , Cifre J , McDowell N , Medrano H , Kaldenhoff R . 2006b. Tobacco aquaporin NtAQP1 is involved in mesophyll conductance to CO_2_ *in vivo* . The Plant Journal 48: 427–439. 17010114 10.1111/j.1365-313X.2006.02879.x

[nph71236-bib-0052] Furbank RT , Foyer CH , Walker DA . 1987. Regulation of photosynthesis in isolated spinach chloroplasts during orthophosphate limitation. Biochimica et Biophysica Acta (BBA) – Bioenergetics 894: 552–561.

[nph71236-bib-0053] Gilbert ME , Pou A , Zwieniecki MA , Holbrook NM . 2012. On measuring the response of mesophyll conductance to carbon dioxide with the variable *J* method. Journal of Experimental Botany 63: 413–425. 21914657 10.1093/jxb/err288 PMC3245476

[nph71236-bib-0054] Gillon JS , Yakir D . 2000. Naturally low carbonic anhydrase activity in C_4_ and C_3_ plants limits discrimination against CO^18^O' during photosynthesis. Plant, Cell & Environment 23: 903–915.

[nph71236-bib-0055] Griffith D , Deutscher N , Caldow C , Kettlewell G , Riggenbach M , Hammer S . 2012. A Fourier transform infrared trace gas and isotope analyser for atmospheric applications. Atmospheric Measurement Techniques 5: 2481–2498.

[nph71236-bib-0056] Hanba YT , Shibasaka M , Hayashi Y , Hayakawa T , Kasamo K , Terashima I , Katsuhara M . 2004. Overexpression of the barley aquaporin HvPIP2; 1 increases internal CO_2_ conductance and CO_2_ assimilation in the leaves of transgenic rice plants. Plant and Cell Physiology 45: 521–529. 15169933 10.1093/pcp/pch070

[nph71236-bib-0057] Harley P , Sharkey T . 1991. An improved model of C_3_ photosynthesis at high CO_2_: reversed O_2_ sensitivity explained by lack of glycerate reentry into the chloroplast. Photosynthesis Research 27: 169–178. 24414689 10.1007/BF00035838

[nph71236-bib-0058] Harley PC , Loreto F , Di Marco G , Sharkey TD . 1992. Theoretical considerations when estimating the mesophyll conductance to CO_2_ flux by analysis of the response of photosynthesis to CO_2_ . Plant Physiology 98: 1429–1436. 16668811 10.1104/pp.98.4.1429 PMC1080368

[nph71236-bib-0059] Holloway‐Phillips M , Cernusak LA , Stuart‐Williams H , Ubierna N , Farquhar GD . 2019. Two‐source *δ* ^18^O method to validate the CO^18^O‐photosynthetic discrimination model: implications for mesophyll conductance. Plant Physiology 181: 1175–1190. 31519787 10.1104/pp.19.00633 PMC6836848

[nph71236-bib-0060] Hommel R , Siegwolf R , Saurer M , Farquhar GD , Kayler Z , Ferrio JP , Gessler A . 2014. Drought response of mesophyll conductance in forest understory species – impacts on water‐use efficiency and interactions with leaf water movement. Physiologia Plantarum 152: 98–114. 24483818 10.1111/ppl.12160

[nph71236-bib-0061] Huber SC . 1989. Biochemical mechanism for regulation of sucrose accumulation in leaves during photosynthesis. Plant Physiology 91: 656–662. 16667083 10.1104/pp.91.2.656 PMC1062051

[nph71236-bib-0062] Jones HG . 1978. Modelling diurnal trends of leaf water potential in transpiring wheat. Journal of Applied Ecology 15: 613–626.

[nph71236-bib-0063] Kitao M , Harayama H , Uemura A . 2017. A practical approach to estimate diffusional leakages of leaf chamber of open gas exchange systems using intact leaves. Plant, Cell & Environment 40: 2870–2874. 10.1111/pce.13032 28984370

[nph71236-bib-0064] Knauer J , Cuntz M , Evans JR , Niinemets Ü , Tosens T , Veromann‐Jürgenson LL , Werner C , Zaehle S . 2022. Contrasting anatomical and biochemical controls on mesophyll conductance across plant functional types. New Phytologist 236: 357–368. 35801854 10.1111/nph.18363 PMC9804998

[nph71236-bib-0065] Kromdijk J , Głowacka K , Long SP . 2020. Photosynthetic efficiency and mesophyll conductance are unaffected in *Arabidopsis thaliana* aquaporin knock‐out lines. Journal of Experimental Botany 71: 318–329. 31731291 10.1093/jxb/erz442

[nph71236-bib-0066] Laisk A . 1983. Calculation of leaf photosynthetic parameters considering the statistical distribution of stomatal apertures. Journal of Experimental Botany 34: 1627–1635.

[nph71236-bib-0067] Laisk A , Eichelmann H . 1989. Towards understanding oscillations: a mathematical model of the biochemistry of photosynthesis. Philosophical Transactions of the Royal Society B 323: 369–384.

[nph71236-bib-0068] Lanigan GJ , Betson N , Griffiths H , Seibt U . 2008. Carbon isotope fractionation during photorespiration and carboxylation in *Senecio* . Plant Physiology 148: 2013–2020. 18923019 10.1104/pp.108.130153 PMC2593675

[nph71236-bib-0069] Leakey AD , Uribelarrea M , Ainsworth EA , Naidu SL , Rogers A , Ort DR , Long SP . 2006. Photosynthesis, productivity, and yield of maize are not affected by open‐air elevation of CO_2_ concentration in the absence of drought. Plant Physiology 140: 779–790. 16407441 10.1104/pp.105.073957 PMC1361343

[nph71236-bib-0070] Lehmeier C , Pajor R , Lundgren MR , Mathers A , Sloan J , Bauch M , Mitchell A , Bellasio C , Green A , Bouyer D *et al*. 2017. Cell density and airspace patterning in the leaf can be manipulated to increase leaf photosynthetic capacity. The Plant Journal 92: 981–994. 28963748 10.1111/tpj.13727 PMC5725688

[nph71236-bib-0071] Lenth RV . 2024. emmeans: estimated marginal means, aka least‐squares means.

[nph71236-bib-0072] Li X‐P , Gilmore AM , Caffarri S , Bassi R , Golan T , Kramer D , Niyogi KK . 2004. Regulation of photosynthetic light harvesting involves intrathylakoid lumen pH sensing by the PsbS protein. Journal of Biological Chemistry 279: 22866–22874. 15033974 10.1074/jbc.M402461200

[nph71236-bib-0073] Loreto F , Di Marco G , Tricoli D , Sharkey TD . 1994. Measurements of mesophyll conductance, photosynthetic electron transport and alternative electron sinks of field grown wheat leaves. Photosynthesis Research 41: 397–403. 24310154 10.1007/BF02183042

[nph71236-bib-0074] Márquez DA , Stuart‐Williams H , Farquhar GD . 2021. An improved theory for calculating leaf gas exchange more precisely accounting for small fluxes. Nature Plants 7: 317–326. 33649595 10.1038/s41477-021-00861-w

[nph71236-bib-0075] McNevin DB , Badger MR , Kane HJ , Farquhar GD . 2006. Measurement of (carbon) kinetic isotope effect by Rayleigh fractionation using membrane inlet mass spectrometry for CO_2_‐consuming reactions. Functional Plant Biology 33: 1115–1128. 32689322 10.1071/FP06201

[nph71236-bib-0076] Medrano H , Escalona JM , Bota J , Gulías J , Flexas J . 2002. Regulation of photosynthesis of C_3_ plants in response to progressive drought: stomatal conductance as a reference parameter. Annals of Botany 89: 895–905. 12102515 10.1093/aob/mcf079 PMC4233802

[nph71236-bib-0077] Miyazawa S‐I , Yoshimura S , Shinzaki Y , Maeshima M , Miyake C . 2008. Deactivation of aquaporins decreases internal conductance to CO_2_ diffusion in tobacco leaves grown under long‐term drought. Functional Plant Biology 35: 553–564. 32688811 10.1071/FP08117

[nph71236-bib-0078] Mizokami Y , Noguchi K , Kojima M , Sakakibara H , Terashima I . 2015. Mesophyll conductance decreases in the wild type but not in an ABA‐deficient mutant (aba1) of Nicotiana plumbaginifolia under drought conditions. Plant, Cell & Environment 38: 388–398. 10.1111/pce.12394 24995523

[nph71236-bib-0079] Mizokami Y , Noguchi K , Kojima M , Sakakibara H , Terashima I . 2019. Effects of instantaneous and growth CO_2_ levels and abscisic acid on stomatal and mesophyll conductances. Plant, Cell & Environment 42: 1257–1269. 10.1111/pce.13484 30468514

[nph71236-bib-0080] Mizokami Y , Oguchi R , Sugiura D , Yamori W , Noguchi K , Terashima I . 2022. Cost–benefit analysis of mesophyll conductance: diversities of anatomical, biochemical and environmental determinants. Annals of Botany 130: 265–283. 35947983 10.1093/aob/mcac100 PMC9487971

[nph71236-bib-0081] Morgan PB , Bollero GA , Nelson RL , Dohleman FG , Long SP . 2005. Smaller than predicted increase in aboveground net primary production and yield of field‐grown soybean under fully open‐air [CO_2_] elevation. Global Change Biology 11: 1856–1865.

[nph71236-bib-0082] Mott KA , Buckley TN . 2000. Patchy stomatal conductance: emergent collective behaviour of stomata. Trends in Plant Science 5: 258–262. 10838617 10.1016/s1360-1385(00)01648-4

[nph71236-bib-0083] Nagler T . 2025. wdm: weighted dependence measures R package version 0.2.6.

[nph71236-bib-0084] Naumann G , Cammalleri C , Mentaschi L , Feyen L . 2021. Increased economic drought impacts in Europe with anthropogenic warming. Nature Climate Change 11: 485–491.

[nph71236-bib-0085] Ogée J , Wingate L , Genty B . 2018. Estimating mesophyll conductance from measurements of C^18^OO photosynthetic discrimination and carbonic anhydrase activity. Plant Physiology 178: 728–752. 30104255 10.1104/pp.17.01031 PMC6181052

[nph71236-bib-0086] Paul MJ , Foyer CH . 2001. Sink regulation of photosynthesis. Journal of Experimental Botany 52: 1383–1400. 11457898 10.1093/jexbot/52.360.1383

[nph71236-bib-0087] Perez‐Martin A , Michelazzo C , Torres‐Ruiz JM , Flexas J , Fernández JE , Sebastiani L , Diaz‐Espejo A . 2014. Regulation of photosynthesis and stomatal and mesophyll conductance under water stress and recovery in olive trees: correlation with gene expression of carbonic anhydrase and aquaporins. Journal of Experimental Botany 65: 3143–3156. 24799563 10.1093/jxb/eru160 PMC4071832

[nph71236-bib-0088] Portis AR Jr . 1992. Regulation of ribulose 1,5‐bisphosphate carboxylase/oxygenase activity. Annual Review of Plant Biology 43: 415–437.

[nph71236-bib-0089] Quirk J , Bellasio C , Johnson DA , Osborne CP , Beerling DJ . 2019. C_4_ savanna grasses fail to maintain assimilation in drying soil under low CO_2_ compared with C_3_ trees despite lower leaf water demand. Functional Ecology 33: 388–398.

[nph71236-bib-0090] R Core Team . 2023. R: a language and environment for statistical computing. Vienna, Austria: R Foundation for Statistical Computing.

[nph71236-bib-0091] Reicosky D , Kaspar T , Taylor H . 1982. Diurnal relationship between evapotranspiration and leaf water potential of field‐grown soybeans. Agronomy Journal 74: 667–673.

[nph71236-bib-0092] Roach T , Krieger‐Liszkay A . 2014. Regulation of photosynthetic electron transport and photoinhibition. Current Protein and Peptide Science 15: 351–362. 24678670 10.2174/1389203715666140327105143 PMC4030316

[nph71236-bib-0093] Rockwell FE , Holbrook NM , Jain P , Huber AE , Sen S , Stroock AD . 2022. Extreme undersaturation in the intercellular airspace of leaves: a failure of Gaastra or Ohm? Annals of Botany 130: 301–316. 35896037 10.1093/aob/mcac094 PMC9486918

[nph71236-bib-0094] Roig‐Oliver M , Bresta P , Nadal M , Liakopoulos G , Nikolopoulos D , Karabourniotis G , Bota J , Flexas J . 2020. Cell wall composition and thickness affect mesophyll conductance to CO_2_ diffusion in *Helianthus annuus* under water deprivation. Journal of Experimental Botany 71: 7198–7209. 32905592 10.1093/jxb/eraa413

[nph71236-bib-0095] Saathoff AJ , Welles J . 2021. Gas exchange measurements in the unsteady state. Plant, Cell & Environment 44: 3509–3523. 10.1111/pce.14178 PMC9292621 34480484

[nph71236-bib-0096] Sade N , Shatil‐Cohen A , Attia Z , Maurel C , Boursiac Y , Kelly G , Granot D , Yaaran A , Lerner S , Moshelion M . 2014. The role of plasma membrane aquaporins in regulating the bundle sheath‐mesophyll continuum and leaf hydraulics. Plant Physiology 166: 1609–1620. 25266632 10.1104/pp.114.248633 PMC4226360

[nph71236-bib-0097] Savage MJ , Cass A . 1984. Psychrometric field measurement of water potential changes following leaf excision. Plant Physiology 74: 96–98. 16663394 10.1104/pp.74.1.96 PMC1066631

[nph71236-bib-0098] Schaeufele R , Santrucek J , Schnyder H . 2011. Dynamic changes of canopy‐scale mesophyll conductance to CO_2_ diffusion of sunflower as affected by CO_2_ concentration and abscisic acid. Plant, Cell & Environment 34: 127–136. 10.1111/j.1365-3040.2010.02230.x 21029117

[nph71236-bib-0099] Scoffoni C , Vuong C , Diep S , Cochard H , Sack L . 2014. Leaf shrinkage with dehydration: coordination with hydraulic vulnerability and drought tolerance. Plant Physiology 164: 1772–1788. 24306532 10.1104/pp.113.221424 PMC3982740

[nph71236-bib-0100] Secchi F , Zwieniecki MA . 2014. Down‐regulation of plasma intrinsic protein1 aquaporin in poplar trees is detrimental to recovery from embolism. Plant Physiology 164: 1789–1799. 24572173 10.1104/pp.114.237511 PMC3982741

[nph71236-bib-0101] Sevanto S . 2018. Drought impacts on phloem transport. Current Opinion in Plant Biology 43: 76–81. 29448177 10.1016/j.pbi.2018.01.002

[nph71236-bib-0102] Sharkey TD . 1985. O_2_‐insensitive photosynthesis in C_3_ plants: its occurrence and a possible explanation. Plant Physiology 78: 71–75. 16664211 10.1104/pp.78.1.71 PMC1064678

[nph71236-bib-0103] Sharkey TD , Badger MR . 1982. Effects of water stress on photosynthetic electron transport, photophosphorylation, and metabolite levels of *Xanthium strumarium* mesophyll cells. Planta 156: 199–206. 24272466 10.1007/BF00393725

[nph71236-bib-0104] Sharkey TD , Seemann JR . 1989. Mild water stress effects on carbon‐reduction‐cycle intermediates, ribulose bisphosphate carboxylase activity, and spatial homogeneity of photosynthesis in intact leaves. Plant Physiology 89: 1060–1065. 16666664 10.1104/pp.89.4.1060 PMC1055975

[nph71236-bib-0105] Smith JAC , Milburn JA . 1980. Phloem turgor and the regulation of sucrose loading in *Ricinus communis* L. Planta 148: 42–48. 24311264 10.1007/BF00385440

[nph71236-bib-0106] Stangl ZR , Tarvainen L , Wallin G , Ubierna N , Räntfors M , Marshall JD . 2019. Diurnal variation in mesophyll conductance and its influence on modelled water‐use efficiency in a mature boreal *Pinus sylvestris* stand. Photosynthesis Research 141: 53–63. 31123952 10.1007/s11120-019-00645-6 PMC6612512

[nph71236-bib-0107] Stitt M . 1986. Limitation of photosynthesis by carbon metabolism 1 Evidence for excess electron transport capacity in leaves carrying out photosynthesis in saturating light and CO_2_ . Plant Physiology 81: 1115–1122. 16664953 10.1104/pp.81.4.1115 PMC1075495

[nph71236-bib-0108] Stitt M , Quick WP . 1989. Photosynthetic carbon partitioning: its regulation and possibilities for manipulation. Physiologia Plantarum 77: 633–641.

[nph71236-bib-0109] Takagi D , Hashiguchi M , Sejima T , Makino A , Miyake CJPR . 2016. Photorespiration provides the chance of cyclic electron flow to operate for the redox‐regulation of P700 in photosynthetic electron transport system of sunflower leaves. Photosynthesis Research 129: 279–290. 27116126 10.1007/s11120-016-0267-5

[nph71236-bib-0110] Tazoe Y , von Caemmerer S , Badger MR , Evans JR . 2009. Light and CO_2_ do not affect the mesophyll conductance to CO_2_ diffusion in wheat leaves. Journal of Experimental Botany 60: 2291–2301. 19255060 10.1093/jxb/erp035

[nph71236-bib-0111] Terashima I , Wong SC , Osmond CB , Farquhar GD . 1988. Characterization of non‐uniform photosynthesis induced by abscisic‐acid in leaves having different mesophyll anatomies. Plant and Cell Physiology 29: 385–394.

[nph71236-bib-0112] Théroux‐Rancourt G , Éthier G , Pepin S . 2014. Threshold response of mesophyll CO_2_ conductance to leaf hydraulics in highly transpiring hybrid poplar clones exposed to soil drying. Journal of Experimental Botany 65: 741–753. 24368507 10.1093/jxb/ert436 PMC3904724

[nph71236-bib-0113] Tikhonov AN . 2014. The cytochrome b6f complex at the crossroad of photosynthetic electron transport pathways. Plant Physiology and Biochemistry 81: 163–183. 24485217 10.1016/j.plaphy.2013.12.011

[nph71236-bib-0114] Trueba S , Pan R , Scoffoni C , John GP , Davis SD , Sack L . 2019. Thresholds for leaf damage due to dehydration: declines of hydraulic function, stomatal conductance and cellular integrity precede those for photochemistry. New Phytologist 223: 134–149. 30843202 10.1111/nph.15779

[nph71236-bib-0115] Ubierna N , Gandin A , Boyd RA , Cousins AB . 2017. Temperature response of mesophyll conductance in three C_4_ species calculated with two methods: ^18^O discrimination and *in vitro* Vpmax. New Phytologist 214: 66–80. 27918624 10.1111/nph.14359

[nph71236-bib-0116] Ubierna N , Gandin A , Cousins AB . 2018a. The response of mesophyll conductance to short‐term variation in CO_2_ in the C_4_ plants *Setaria viridis* and *Zea mays* . Journal of Experimental Botany 69: 1159–1170. 29474683 10.1093/jxb/erx464 PMC6018935

[nph71236-bib-0117] Ubierna N , Holloway‐Phillips M‐M , Farquhar GD . 2018b. Using stable carbon isotopes to study C_3_ and C_4_ photosynthesis: models and calculations. In: Covshoff S , ed. Photosynthesis: methods and protocols. New York, NY, USA: Springer, 155–196. 10.1007/978-1-4939-7786-4_10 29978402

[nph71236-bib-0118] Vassey TL , Sharkey TD . 1989. Mild water stress of *Phaseolus vulgaris* plants leads to reduced starch synthesis and extractable sucrose phosphate synthase activity 1. Plant Physiology 89: 1066–1070. 16666665 10.1104/pp.89.4.1066 PMC1055976

[nph71236-bib-0120] Vrábl D , Vašková M , Hronková M , Flexas J , Šantrůček J . 2009. Mesophyll conductance to CO_2_ transport estimated by two independent methods: effect of variable CO_2_ concentration and abscisic acid. Journal of Experimental Botany 60: 2315–2323. 19433478 10.1093/jxb/erp115

[nph71236-bib-0121] Walker D . 1992. Concerning oscillations. Photosynthesis Research 34: 387–395. 24408834 10.1007/BF00029813

[nph71236-bib-0122] Weerasooriya HN , Longstreth DJ , DiMario RJ , Rosati VC , Cassel BA , Moroney JV . 2024. Carbonic anhydrases in the cell wall and plasma membrane of *Arabidopsis thaliana* are required for optimal plant growth on low CO_2_ . Frontiers in Molecular Biosciences 11: 1267046. 38455761 10.3389/fmolb.2024.1267046 PMC10917985

[nph71236-bib-0123] Xu D‐Q , Shen Y‐K . 1996. Midday depression of photosynthesis. In: Handbook of photosynthesis. Boca Raton, FL, USA: CRC Press, 451–459.

[nph71236-bib-0124] Zhou Y , Lam HM , Zhang J . 2007. Inhibition of photosynthesis and energy dissipation induced by water and high light stresses in rice. Journal of Experimental Botany 58: 1207–1217. 17283375 10.1093/jxb/erl291

