# Supplementary material for: Rapid dehydration drives a nondiffusional drop in C3 photosynthesis that aligns with phosphate limitation
Source: New Phytol. 2026 May 18;251(3):1127–42. doi: 10.1111/nph.71236 (PMC13326525; doi:10.1111/nph.71236)
Supplement: Supplementary file 2 — Fig. S1 Example of pressure–volume curve obtained for wheat. Fig. S2 Experimental Set‐up. Fig. S3 Relationship between gas‐exchange variables and leaf water potential. Fig. S4 Fluorescence and isotope discrimination variables in relation to water potential. Fig. S5 The relationship between net assimilation and mesophyll conductance estimated by three different methods. Fig. S6 Sensitivity analysis of the effect of cuticular conductance, unsaturation and leaf temperature on the differences in mesophyll conductance between hydrated and dehydrated leaves. Methods S1 Hydromechanical characterisation. Methods S2 Accounting for cuticular conductance and unsaturation in the calculation of gas‐exchange variables. Methods S3 Modelling gas exchange on hydrated plants. Methods S4 Modelling mesophyll conductance from given values of limitations. Notes S1 Uncertainties in stomatal transpiration, airspace unsaturation, and leaf temperature. Notes S2 Ψ L heterogeneity. Notes S3 Stomatal patchiness. Notes S4 Oxygen Isotopic Equilibrium. Table S1 Abbreviations, definitions and units for variables and acronyms described in the text. Please note: Wiley is not responsible for the content or functionality of any Supporting Information supplied by the authors. Any queries (other than missing material) should be directed to the New Phytologist Central Office. [file NPH-251-1127-s001.pdf]

Rapid dehydration drives a non-diffusional drop in C_3 photosynthesis that aligns with phosphate limitation

Chandra Bellasio, Daniel Tholen, Hilary Stuart-Williams, Graham D Farquhar, and Jaume Flexas

Supplementary Information File S1:

Note S1.	Uncertainties in stomatal transpiration, airspace unsaturation, and leaf temperature
Note S2.	Ψ_L heterogeneity
Note S3.	Stomatal patchiness
Note S4.	Oxygen Isotopic Equilibrium
Method S1.	Hydromechanical characterisation
Method S2:	Accounting for cuticular conductance and unsaturation in the calculation of gas-exchange variables
Method S3.	Modelling gas exchange on hydrated plants
Method S4.	Modelling mesophyll conductance from given values of limitations
Table S1.	Abbreviations, definitions and units for variables and acronyms described in the text
Figure S1.	Example of pressure-volume curve obtained for wheat
Figure S2.	Experimental Setup
Figure S3.	Relationship between gas-exchange variables and leaf water potential
Figure S4.	Fluorescence and isotope discrimination variables in relation to water potential
Figure S5.	The relationship between net assimilation and mesophyll conductance estimated by three different methods.
Figure S6:	Sensitivity analysis of the effect of cuticular conductance, unsaturation and leaf temperature on the differences in mesophyll conductance between hydrated and dehydrated leaves.

Supporting Notes

Note S1. Uncertainties in cuticular transpiration, airspace unsaturation, and leaf temperature

Dehydration amplifies the impact of uncertainties due to reduced gas-exchange fluxes. We investigated whether such uncertainties could explain the observed decrease in g_M.

A first uncertainty is whether all transpiration occurs through the stomata, as conventionally assumed. Márquez *et al.* (2021) showed that ignoring the small transpiration of water through the cuticle may lead to C_i overestimation when stomata close. If the measured transpiration (E) is occurring only in part through stomata, stomatal conductance to water (g_{Sw}) will be lower than total leaf conductance to water. Thus, if this transpiration through the cuticle is ignored, g_{Sw} is overestimated. Since the conductance to CO_2 scales linearly with that of water, and C_i is calculated from stomatal conductance (see Methods S4, Equation S6), then the true C_i would be lower than calculated. This overestimation of C_i for a given C_M may lead to an apparent reduction in $g_M \left(= \frac{A}{C_i - C_M}\right)$ when g_{Sw} decreases. To mitigate such bias, we accounted for a realistic cuticular conductance of 5 mmol m^{-2} s^{-1} in all our analyses, but this did not appreciably affect results. In addition, we conducted a sensitivity analysis to variations in cuticular conductance at the Ψ_L that resulted in approximately a 50% reduction in g_M, and expressed the g_M response relative to the g_M obtained under full hydration. The effect on g_M was minimal (Figure S6) between 0 and 10 mmol m^{-2} s^{-1}, which corresponds to the range typically reported in the literature *e.g.* Slot *et al.* (2021); Márquez *et al.* (2022).

A second uncertainty relates to the intercellular airspace relative humidity (h_i) which cannot be directly measured. The conventional assumption is that h_i is 100 %. If, in reality, h_i is lower, the true humidity gradient across stomata (w_i - w_a; see Eqn 15) is lower than that occurring when h_i is 100 % (w_s - w_a). The measured transpiration is thus attained with a true g_{Sw} that is higher than that estimated, meaning that the true C_i is also higher than that estimated (Cernusak et al., 2024; Tholen, 2024). Recent estimates for h_i were as low as 70 % when mole fraction difference (D_S) rose above ~20 mmol mol^{-1} (Cernusak et al., 2018; Wong et al., 2022; Diao et al., 2024). Such low h_i implies a Ψ_L difference of over 45 MPa between the cytosol and adjacent apoplast, which is unrealistic (Buckley & Sack, 2019). The low estimates may stem from modelling simplifications and assumptions, such as constant airspace g_M, (Wong et al., 2022) or constant ${}^{O}g_M$ (Cernusak et al., 2018), which may be problematic (Holloway-Phillips et al., 2019; Cernusak et al., 2024; Rockwell, 2024). By using CO_2 of two different isotopic compositions Holloway-Phillips et al. (2019) elegantly

avoided assuming invariant $^{O}g_M$, and found h_i remained above 94 %, even when D_S exceeded 20 hPa. Infiltration of the apoplast with hydrogel-based nanoreporters showed that apoplastic Ψ_L in dehydrating stomata leaves remained above -1.6 MPa during atmospheric and soil dry-down experiments, corresponding to <2 % decrease in h_i Jain et al. (2024). Therefore, for experiments done at low D_S, only a moderate decrease in h_i between 90 and 100% seems reasonable. In line with these findings, we found no evidence for h_i decreasing below 90% (Figure 5). These estimates are further supported by the observation that assuming h_i below 90% led to negative g_{Sw} for many data points. We examined whether neglecting the decrease in h_i could have explained the observed changes in g_M with a sensitivity analysis. The response of g_M to dehydration became somewhat more pronounced at progressively lower h_i (Figure S6), but did not significantly alter our conclusions.

A third uncertainty involves the measurement of leaf temperature, which can be influenced by leaf chamber design and the method of thermocouple application (Mott & Peak, 2011). Despite regular calibration against the chamber thermistor, the thermocouple may not perfectly reflect leaf temperature. As transpiring leaves are generally cooler than the air, and the thermocouple measures a blend of both (Mott & Peak, 2011), the leaf temperature values could be overestimated. If the true leaf temperature is lower than measured, the true water vapour pressure in the leaf is lower than what is calculated. This effect is similar to that of airspace unsaturation, but it becomes more pronounced at higher temperatures due to the exponential relationship between temperature and saturated vapour pressure. We examined whether a systematic error in leaf temperature measurement could explain the observed decrease in g_M using a third sensitivity analysis, conducted at the Ψ_L corresponding to approximately a 50% reduction in g_M. Figure S6 shows that the decrease in g_M would largely disappear if the true leaf temperature were at least 1°C higher than measured, which would be rather extreme. Further, in our data, the thermocouple-measured leaf temperature was generally lower than the thermistor-measured air temperature. Based on the regression reported by Garen *et al.* (2022), this would imply that actual leaf temperature was lower than measured – opposite to the error needed to offset the observed g_M decrease. We therefore conclude that temperature measurement error did not affect our conclusions.

Note S2. Ψ_L heterogeneity

To minimise spatial gradients in Ψ_L that can develop across leaves under faster dehydration methods, such as bench-drying (Blackman *et al.*, 2009; Trueba *et al.*, 2019), we gradually withdrew roots from the hydroponic solution. This resulted in a step-wise decrease in Ψ_L leading to steady-state conditions for gas-exchange and isotopic measurements. Water vapour and CO_2 exchanged by leaves were measured in real time at high resolution until leaves were wilted.

The pressure chamber measurements, used in our previous experiments, present the disadvantage of being destructive and cannot be made continuously. Sunflower leaves, being broader than the pressure chamber gasket, would need to be cut into longitudinal strips, potentially introducing bubbles from the cuts that could confound the detection of the equilibrium point.

Psychrometry has two main potential sources of error. First, there may be lateral heterogeneity in water potential between the site of Ψ_L measurement and the site of gas exchange. The thermocouple is sealed to the leaf surface, locally suppressing transpiration and allowing the psychrometer to equilibrate with water in the vein xylem rather than in the mesophyll of actively transpiring tissue. Thus, our Ψ_L measurements should be interpreted as estimates of xylem water potential, and by extension, provide insights about the hydration status of the leaf. If most of the hydraulic resistance is outside the xylem, differences in water potential between xylem and mesophyll could arise, potentially creating a discrepancy between the measured Ψ_L and the actual water potential at the site of gas exchange. Second, the method requires cutting a window in the epidermis, which may cause some cell sap to spill and locally lower the osmotic potential at the measurement site, leading to an overestimation of Ψ_L. We mitigated this risk by rinsing and blotting the exposed tissue repeatedly and allowing the leaf to recover overnight before measurements, promoting osmolyte recapture by viable cells. Furthermore, to prevent systematic bias, the turgor loss point was determined using the same epidermal window technique and validated by curve-fitting of pressure–volume data.

Note S3. Stomatal patchiness

It has been reported that under high D_S or dehydration stomata aperture can become patchy, meaning the size of the apertures varies over the leaf, and may even become bimodal with stomata either fully open or fully closed (Laisk, 1983; Terashima *et al.*, 1988; Mott & Buckley, 2000; Rockwell *et al.*, 2022). This could affect the calculated g_M because C_i estimates from gas exchange are conductance-weighted values (Farquhar *et al.*, 1989; Rockwell *et al.*, 2022). This means that areas of the leaf with open stomata contribute more to the measured C_i, while areas with closed stomata contribute very little or nothing, and thus the contribution of leaf patches with closed stomata to the C_i estimate is minimal. By contrast, estimates for C_M are electron-transport-weighted for the fluorescence method, or assimilation-weighted for isotopic methods (Farquhar *et al.*, 1989) Typically, for instance, the fluorescence signal includes contributions from all parts of the leaf, even where stomata are closed, and thus also represent leaf patches with closed stomata. Combining such differentially weighted estimates for C_i, C_M and A, to calculate g_M may lead to an apparent reduction when patchiness changes during dehydration (Laisk, 1983; Farquhar *et al.*, 1989; Rockwell *et al.*, 2022).

Patchiness is difficult to quantify or to account for during analysis (Laisk, 1983; Pospíšilová & Šantrůček, 1994; Márquez *et al.*, 2023). Downton et al. (1988) found large effects but these may have been overestimated by incorrectly assuming that any decrease in Y(II) during dehydration was solely due to a reduction in C_i, thus neglecting the contribution of L_{NS}. In a comprehensive theoretical study, Buckley et al. (1999) concluded that the impact of patchiness was often minimal, especially when stomatal aperture followed a normal distribution rather than bimodal, and when leaves were 'highly coupled' to the surrounding air. These conditions were likely present in our measurements, where g_S was relatively high (Figure 4C and 4D), boundary layer conductance and thermal gradients were minimized due to vigorous ventilation, transpiration was relatively low (around 3 mmol m^{-2} s^{-1}), and irradiance in the IRGA leaf cuvette was moderate (below 900 µmol m^{-2} s^{-1}). Further, circumstantial evidence suggests that patchiness either did not occur or had an insignificant impact on our findings. Firstly, stomatal patchiness is typically associated with fluctuations in C_i measured by the IRGA (Mott & Buckley, 1998), which we did not observe. Secondly, if stomatal patchiness were to cause a significant misestimation of C_i, then the incipient point of response of L_{NS} would depend on the C_i under which L_{NS} were derived, but we did not find such a difference. Thirdly, the slope of responses to dehydration would be influenced by C_i, but this was not the case either.

Note S4. Oxygen Isotopic equilibrium

Though interpretation and calculations for ${}^{O}g_{M}$ are still debated (Ogée et al. 2018), ${}^{O}g_{M}$ is proposed to reflect the overall capacity of the leaf to deliver CO_2 to the site where it reaches full isotopic equilibrium with water. The oxygen-isotope exchange between water and CO_2 is catalysed by carbonic anhydrases, and the site of equilibrium is hypothesised to be in the mesophyll cytosol (Gillon & Yakir, 2000a). our results support the view that ${}^{O}g_{M}$ captures mesophyll conductance responses, with values consistent with a shorter diffusion pathway compared with ${}^{C}g_{M}$ in the two C_3 species studied.

Until recently, calculating ${}^{O}g_{M}$ required assuming full isotope equilibration. Incomplete isotope equilibrium has been suggested in some species (Gillon & Yakir, 2000b), particularly those with low carbonic anhydrase activity, either occurring naturally, such as in C_4 grasses (Cousins *et al.*, 2008) or in mutant C_3 and C_4 plants (Williams *et al.*, 1996; Cousins *et al.*, 2006). Allowing for incomplete isotope equilibrium when calculating ${}^{O}g_{M}$ would increase estimates of ${}^{O}g_{M}$ (Ubierna *et al.*, 2017; Ogée *et al.*, 2018) and hence the offset with ${}^{C}g_{M}$.

Ogée *et al.* (2018) proposed calculations of ${}^{O}g_{M}$ that do not require assumptions about the degree of isotope equilibrium. However, when Ogée et al. (2018) recomputed previously published datasets using this approach, they found that CO_2 was close to full equilibration in the analysed data. In addition, comparison of ${}^{O}g_{M}$ with g_M derived from independent methods suggested full or near-full isotope equilibrium in three C_4 species (Ubierna *et al.*, 2017).

If the degree of isotope equilibrium varied with dehydration, interpretation of ${}^{O}g_{M}$ could be complicated. For example, a low ${}^{O}g_{M}$ values at more negative Ψ_L, may be actually be inflated when we would assume isotope disequilibrium.

Supporting Methods

Method S1. Hydromechanical characterisation

A small window of epidermis was removed from a fully expanded leaf of a plant standing in aerated water, and then fitted with a calibrated PSY1 psychrometer (ICT, Armidale, Australia). After an hour, when Ψ_L was constant, the leaf was cut and placed on a balance together with the PSY1 mount. Weight and Ψ_L were measured every 10-20 min throughout the day, then the leaf was removed and dried to calculate the relative water content (*RWC*). The apoplastic water fraction (*awf*, fraction of leaf water not contained by the plasmalemma) and the bulk elastic modulus (ε, stiffness of cell walls) were estimated through curve fitting to the experimental values of Ψ_L and *RWC* as *per* Bartlett *et al.* (2012). An example is shown in Figure S1, the fitted values are in Table 1. The bulk osmotic potential ($\Psi_{S\,Bulk}$) was obtained by squeezing thawed leaves onto 5 mm-diameter filter paper discs, measured after 1 hour, as *per* Quirk *et al.* (2019). The osmotic potential at full hydration ($\Psi_{S\,0}$) was (Bartlett *et al.*, 2012):

$$\Psi_{S\,0} = \Psi_{S\,Bulk}\left(1 + \frac{awf}{100}\right). \qquad S1$$

Finally, the water potential at turgor loss Ψ_{TL} was (Bartlett *et al.*, 2012):

$$\Psi_{TL} = \frac{\Psi_{S\,0}\,\varepsilon}{\Psi_{S\,0} + \varepsilon}. \qquad S2$$

Bulk elastic modulus (ε) obtained by fitting pressure volume curves was on average 7.25 ± 0.75 (SE) and 4.07 ± 0.66 MPa, while the apoplastic water fraction (*awf*) was on average 0.002 ± 0.027 and 0.103 ± 0.028 g g^{-1} for n=7 wheat and sunflower, respectively.

Method S2: Accounting for cuticular conductance and unsaturation in the calculation of gas-exchange variables

Following Márquez *et al.* (2021), the transpirational flux through the cuticle (E_c) can be calculated as:

$$E_c = g_{cw}(w_i - w_a)\ , \qquad S3$$

where w_i represents the water vapour mole fraction in the substomatal air spaces, and e_a is the water vapour mole fraction in the gas-exchange chamber. w_i is calculated as $h_i\,w_s$, where h_i is the assumed substomatal relative humidity and w_s is the saturation water vapour mole fraction. The flux through the stomata (E_s) is then simply:

$$E_s = E - E_c\,, \qquad S4$$

where E is the measured transpiration. Total conductance to water vapor is then calculated by modifying Eqn 15 as per Eqn 10 in Márquez *et al.* (2021):

$$g_{\mathrm{tw}} = \left(\frac{w_{\mathrm{i}} - w_{\mathrm{a}}}{E - \frac{1}{2}E_{\mathrm{s}}(w_{\mathrm{i}} + w_{\mathrm{a}})} + \frac{1}{g_{\mathrm{bw}}}\right)^{-1}, \tag{S5}$$

where g_{bw} is the boundary layer conductance to water vapour as given by the LI6400XT. The stomatal conductance to water vapour can now be calculated by rearranging Eqn 1 in Supplemental note 3 in Márquez *et al.* (2021) as:

$$g_{\mathrm{Sw}} = \left(\frac{1}{g_{\mathrm{tw}}} - \frac{1}{g_{\mathrm{bw}}}\right)^{-1} - g_{\mathrm{cw}}, \tag{S6}$$

The CO_2 mole fraction at the leaf surface was calculated as per Eqn 12 in Márquez *et al.* (2021):

$$C_{\mathrm{s}} = \frac{g_{\mathrm{bc}}\, C_{\mathrm{a}} - A - \frac{E}{2} C_{\mathrm{a}}}{g_{\mathrm{bc}} + \frac{E}{2}}, \tag{S7}$$

where g_{bc} is the boundary layer conductance to CO_2 $\left(g_{\mathrm{bc}} = \frac{g_{\mathrm{bw}}}{1.37}\right)$.

The mole fraction of substomatal CO_2 can now be calculated as per Eqn 13 in Márquez *et al.* (2021):

$$C_{\mathrm{i}} = \frac{C_{\mathrm{s}}(g_{\mathrm{SC}} + g_{\mathrm{cc}} - \frac{E_{\mathrm{S}}}{2}) - A}{g_{\mathrm{SC}} + g_{\mathrm{cc}} + \frac{E_{\mathrm{S}}}{2}}, \tag{S8}$$

where g_{SC} is the stomatal conductance to CO_2 $\left(g_{\mathrm{SC}} = \frac{g_{\mathrm{Sw}}}{1.6}\right)$, and g_{cc} is the cuticular conductance to CO_2, here assumed to be 0.025 g_{cw}.

Method S3. Modelling gas exchange on hydrated plants

The relationship between A and C_i was modelled empirically as (Bellasio *et al.*, 2016):

$$A_{\mathrm{mod}} = \frac{CE(C_{\mathrm{i}} - \Gamma) + A_{\mathrm{SAT}} - \sqrt{(CE[C_{\mathrm{i}} - \Gamma] + A_{\mathrm{SAT}})^2 - (4\omega A_{\mathrm{SAT}} CE[C_{\mathrm{i}} - \Gamma])}}{2\omega}, \tag{S9}$$

where A_{SAT} represents the CO_2-saturated rate of A under the *PPFD* of the measurements; *CE* represents carboxylation efficiency, ω is an empirical factor defining curvature; and Γ is the x–intercept, *i.e.* the C_i at which A_{mod} is zero.

The dependence of gross assimilation ($GA = A + R_{LIGHT}$) on *PPFD* was modelled as (Prioul & Chartier, 1977):

$$GA = \frac{Y(CO_2)_{\mathrm{LL}}\, PPFD + GA_{\mathrm{SAT}} - \sqrt{(Y(CO_2)_{\mathrm{LL}}\, PPFD + GA_{\mathrm{SAT}})^2 - 4\, m\, Y(CO_2)_{\mathrm{LL}}\, PPFD\; GA_{\mathrm{SAT}}}}{2\, m}, \tag{S10}$$

where GA_{SAT} represents the light-saturated rate of GA under the [CO_2] of the measurements; $Y(CO_2)_{LL}$ is the quantum yield for CO_2 fixation; m is an empirical factor defining curvature. These parameters were estimated together with R_{LIGHT} by fitting Eqn S9 and S10.

Method S4. Modelling mesophyll conductance from given values of limitations

The dependence of A upon C_i was modelled through Eqn S9 and parameterized with the average fitted photosynthetic parameters shown in Table 1. The potential assimilation that would occur in fully hydrated leaves (A_p) if the substomatal cavity were directly exposed to C_a was calculated by substituting C_a for C_i in Eqn S9. Stomatal limitation (L_S) reduces assimilation to a point A_h, which is $A_h = A_p\,(1 - L_S)$. To calculate the corresponding C_i, the A/C_i curves passing for A_h are found by solving Eqn S9 for C_i, as:

$$C_i = \frac{\omega A_p{}^2 + CE \quad \Gamma A_p - A_{SAT}{}^2 A_p - A_{SAT} \quad CE \quad \Gamma}{CE \quad A_p - A_{SAT} \quad CE}. \qquad \text{S11}$$

Non-stomatal limitation (L_{NS}) may reduce assimilation to a point A_d, which is $A_d = A_h - A_p\,L_{NS}$. If non-stomatal limitation is solely caused by a reduction in g_M, then the reduction from A_h to A_d is entirely caused by a difference in of CO_2 mole fraction between C_i and at the mesophyll carboxylating sites C_M. The pairs (C_M, A_d) would then trace the hydrated A/C_M curve, therefore C_M corresponding to A_d can be obtained using Eqn S11 parameterized using CE and ω fitted on A/C_M curves (shown in Table 1). Finally, stomatal and mesophyll conductances to CO_2 are $g_{SC} = \frac{A_d}{C_a - C_i}$, and $g_M = \frac{A_d}{C_i - C_M}$.

Table S1. Abbreviations, definitions and units for variables.

Symbol	Definition	Values/Units
a	Combined ^{13}C fractionation for diffusion of CO across the stomata and boundary layers. $a = \frac{a_b(C_a-C_b)+a_s(C_b-C_i)}{C_a-C_i}$, where C_b is the CO_2 mole fraction in the boundary layer	a_b = 2.9 (Farquhar, 1983), a_s = 4.4 ‰ (Craig, 1953)
A	Net assimilation at ambient O_2	µmol m^{-2} s^{-1}
a^{18}	Combined ^{18}O fractionation for diffusion of CO_2 across the stomata and boundary layer $a^{18} = \frac{a_b^{18}\ (C_a-C_b)+a_s^{18}(C_b-C_i)}{C_a-C_i}$, where C_b is the CO_2 mole fraction in the boundary layer	a_b^{18}= 5.8‰; a_s^{18}= 8.8 ‰; Farquhar and Lloyd (1993)
A_d	Modelled assimilation of dehydrated leaves at C_i	µmol m^{-2} s^{-1}
A_h	Modelled assimilation of hydrated leaves at C_i	µmol m^{-2} s^{-1}
A_{Low}	Net assimilation rate measured at an O_2 mole fraction of 20 mmol mol^{-1}	
a_m^{18}	^{18}O fractionation during liquid phase diffusion and dissolution	0.8‰; Farquhar and Lloyd (1993)
a_m	^{13}C fractionation associated with diffusion through liquid	1.8 ‰ (O'Leary, 1984)
A_p	Potential assimilation of hydrated leaves if C_i = C_a	µmol m^{-2} s^{-1}
A_{SAT}	CO_2-saturated rate of A under the *PPFD* of the measurements	µmol m^{-2} s^{-1}
awf	Fraction of apoplastic water (Bartlett *et al.*, 2012)	%
b	^{13}C fractionation during carboxylation	30 ‰ (Roeske & Oleary, 1984; McNevin *et al.*, 2006)
β	Ratio between cuticular conductance to CO_2 and to water	0.025 (Márquez *et al.* (2021)
C_a	The CO_2 mole fraction in the cuvette as measured by IRGA	µmol mol^{-1}
C_{CA}	The CO_2 mole fraction at the site of the oxygen isotopic equilibrium	µmol mol^{-1}
CE	Initial slope of the modelled A/Ci curve	mol m^2 s^{-1}
C_i	The CO_2 mole fraction in the sub-stomatal cavity	µmol mol^{-1}
C_{in}, C_{out}	The $^{12}CO_2$ mole fraction entering and leaving the leaf chamber	µmol mol^{-1}
C_S	The CO_2 mole fraction at the leaf surface	µmol mol^{-1}
$\delta^{13}C$	Isotopic composition ($^{18}C/^{16}C$) of CO_2 relative to Pee Dee Belemnite	‰
Δ_o^{18}	Observed isotope discrimination against ^{18}O	‰
$\delta^{18}O$	Isotopic composition ($^{18}O/^{16}O$) relative to Vienna Standard Mean Ocean Water	‰
δ_A^{18}	$\delta^{18}O$ of the CO_2 taken up by photosynthesis	‰
δ_{A}^{18}	$\delta^{18}O$ of the CO_2 taken up by photosynthesis (Cernusak et al., 2004)	‰
$\Delta_{control}$	Observed discrimination against ^{13}C, measured under ambient growth conditions	-22 ‰
δ_{ca}^{18}	$\delta^{18}O$ of CO_2 at the sites of carbonic anhydrase activity	‰; Eqn 16
δ_{ce}^{18}	$\delta^{18}O$ of CO_2 in equilibrium with cytosol water	‰
δ_i^{18}	$\delta^{18}O$ of CO_2 in the substomatal cavity	‰; Eqn 11
Δ_i	The modelled ^{13}C isotope discrimination when g_M is assumed to be infinite (Busch et al. 2020)	‰; Eqn 9
δ_{in}^{18} ,δ_{out}^{18}	$\delta^{18}O$ of the CO_2 in the air entering and leaving the leaf chamber	‰
Δ_o	Observed isotope discrimination against ^{13}C	‰
D_S	Water mole fraction difference between inside and outside the leaf	mmol mol^{-1}
δ_{w-e}^{18}	$\delta^{18}O$ of liquid water at evaporative sites in leaves	‰
δ_{w-E}^{18}	The δ18O of water vapour transpired by the leaf (Craig et al., 1965)	‰; Eqn 14
δ_{w-in}^{18} , δ_{w-out}^{18}	$\delta^{18}O$ of the H_2O in the air entering and leaving the leaf chamber	‰
e	^{13}C fractionation associated with respiratory CO_2 release.	-3 ‰; (Bellasio & Griffiths,
E	Measured transpiration rate	mmol m^{-2} s^{-1}
ε	Bulk elastic modulus of the cell (Bartlett *et al.*, 2012)	MPa
$e´$	Apparent fractionation representing the combined effect of e and that of the release of respiratory substrates previously fixed under growth conditions	‰
ε^+	Equilibrium ^{18}O fractionation between liquid water and vapour	‰

E_c	Transpiration through the cuticle	mmol m^{-2} s^{-1}
ε_K	kinetic fractionation during diffusion of $H_2{}^{18}O$ from the substomatal air spaces to the atmosphere	‰
$\eta(A)_{O2}$	The relative sensitivity (elasticity) of assimilation to 2% O_2	dimensionless
$\eta(YII)_{O2}$	The relative sensitivity (elasticity) of the yield of photosystem II to 2% O_2	dimensionless
ε_w	Equilibrium ^{18}O fractionation between CO_2 and water	‰
f	^{13}C fractionation during photorespiration.	-11.6 ‰ (Lanigan *et al.*, 2008).
GA	Gross assimilation (chloroplast CO_2 consumption), $GA = A + R_{Light}$	μmol m^{-2} s^{-1}
Γ	C_i–A compensation point, i.e. C_i at which A=0 and $V_C = R_{Light} + F$	μmol mol^{-1}
Γ^*	Half the reciprocal of the Rubisco specificity times O_2 mole fraction	39.7 in wheat and 37 μmol mol^{-1} in sunflower at 21% O_2
GA_{SAT}	Light-saturated gross assimilation rate	μmol m^{-2} s^{-1}
g_{bc}	Boundary layer conductance to CO_2	mol m^{-2} s^{-1}
g_{bw}	Boundary layer conductance to water vapour	mol m^{-2} s^{-1}
g_{cw}	Cuticular conductance to water vapour	mol m^{-2} s^{-1}
g_M, ${}^Jg_M$ ${}^Cg_M$, ${}^Og_M$	Mesophyll conductance to CO_2, generic, derived from electron transport rate, carbon or oxygen discrimination, respectively	mol m^2 s^{-1}
g_{SC}	Stomatal conductance to CO_2	mol m^2 s^{-1}
g_{sw}	Stomatal conductance to water vapour	mol m^{-2} s^{-1}
g_{tw}	Total conductance to water vapour	mol m^{-2} s^{-1}; Eqn 15
h_i	Substomatal relative humidity	dimensionless
IRGA	Infrared gas analyser	
j, j_{ATP}	Electron transport rate, ATP production rate	μmol m^{-2} s^{-1}
L_S, L_{NS}	Stomatal and non-stomatal limitation (Bellasio *et al.*, 2018; Bellasio, 2025)	dimensionless
m	Curvature of the non-rectangular hyperbola fitted to describe the *PPFD* dependence of *GA*	dimensionless
n	Number of biological replicates	dimensionless
O_M	O_2 mol fraction in the mesophyll cells (in air at equilibrium)	210 mmol mol^{-1}
ω	Curvature of the non-rectangular hyperbola describing the C_i dependence of A	dimensionless
P	Atmospheric pressure	Pa
ϕ	The ratio of the Rubisco oxygenation rate relative to its carboxylation rate V_O / V_C)	dimensionless
PPFD	Photosynthetic photon flux density	μmol m^{-2} s^{-1}
$\mathscr{q}$	Electrons or ATP required by gross assimilation under low $[O_2]$	e^- or ATP / CO_2
R_{Light}	Total non-photorespiratory CO_2 production in the light	μmol m^{-2} s^{-1}
Rubisco	Ribulose bisphosphate carboxylase oxygenase	
RWC	Relative water content	%
t, t^{18}	Ternary correction factors dependent on the rate of transpiration (Farquhar & Cernusak, 2012)	dimensionless
V_C	Rubisco carboxylation rate	μmol m^{-2} s^{-1}
V_O	Rubisco oxygenation rate	μmol m^{-2} s^{-1}
w_a	The H_2O mole fraction in leaf chamber	mmol mol^{-1}
w_i	The H_2O mole fraction in the substomatal cavity	mmol mol^{-1}
w_{in}, w_{out}	The H_2O mole fraction in the air entering and leaving the leaf chamber	μmol mol^{-1}
w_s	The saturation H_2O mole fraction at leaf temperature and air pressure	mmol mol^{-1}
$Y(II)$	Yield of photosystem II $Y(II) = \frac{F'_m - F_s}{F'_m}$ (Genty *et al.*, 1989)	dimensionless
$Y(CO_2)_{LL}$	Initial slope of the modelled light curve (quantum yield)	mol CO_2 (mol photons)$^{-1}$
Ψ_L	Leaf water potential	MPa
$\Psi_{S\ Bulk}$	Bulk osmotic potential	MPa
$\Psi_{S\ 0}$	Osmotic potential at full hydration	MPa
Ψ_{TL}	Water potential at turgor loss	MPa

Figure S1. Example of pressure-volume curve obtained for wheat

Pressure volume curves were constructed by concurrently measuring leaf relative water content (*RWC,* gravimetrically) and leaf water potential (Ψ_L, with a psychrometer). Triangles show measured values and the line the model fitted the data, which was used to estimate the apoplastic water fraction (*awf*) and the bulk elastic modulus (ε, values in Table 1).

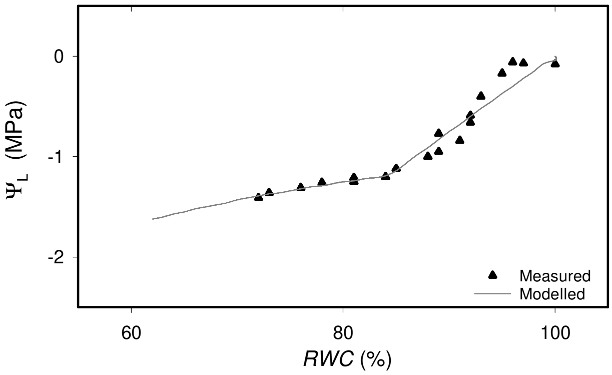

Figure S2. Experimental Setup for concurrent measurements of gas exchange, fluorometry, oxygen and carbon isotopic discrimination

1, gas mixing unit; 2, dew point control; 3, gas-exchange analyser; 4, light; 5, plant; 6, water level regulator; 7, fluorometer (not visible in B); 8, Aerodyne Carbon Dioxide Isotope Monitor (in a thermostatic assembly); 9, Picarro Ring-Down Spectrometer; 10, Psychrometer (not visible); 11 water vapour calibration device.

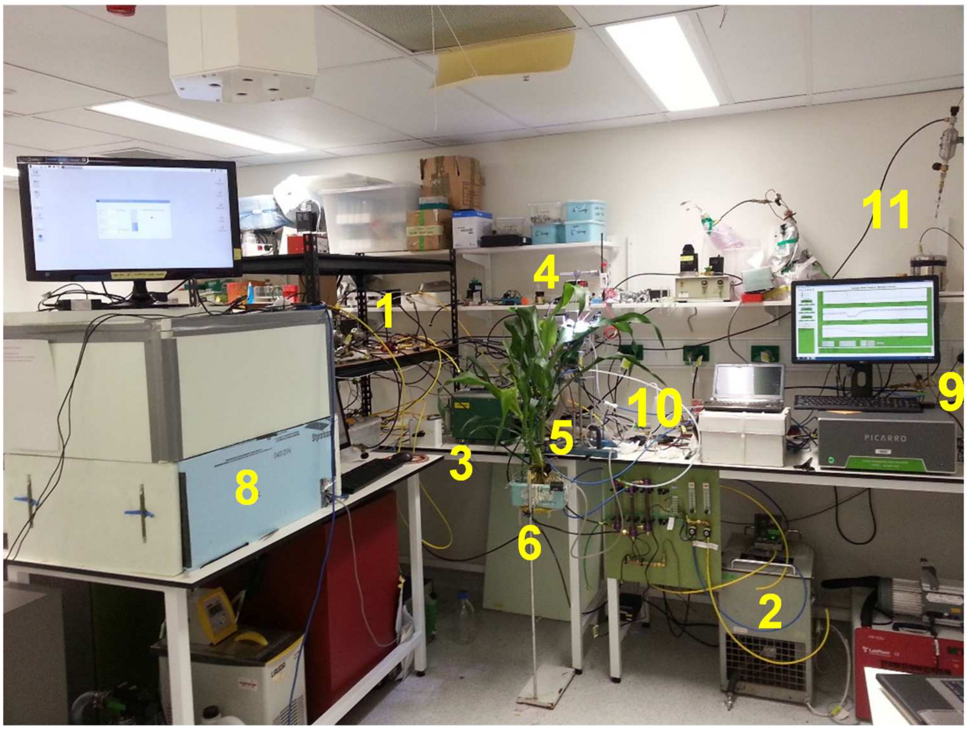

Figure S3. Gas exchange under rapid dehydration.
Wheat (left) and sunflower (right) plants grown on hydroponics were progressively pulled out of the water while water potential (Ψ_L) and gas exchange were measured every ten minutes. Panels **A** and **B**, assimilation; Panels **C** and **D**, stomatal conductance (g_S); Panels **E** and **F**, CO_2 mole fraction in the substomatal cavity (C_i); Panels **G** and **H**, stomatal limitation (L_S), Panels **I** and **J**, non-stomatal limitation (L_{NS}). L_S and L_{NS} were calculated using the parameters in Table 1 obtained by curve fitting to the measured A/C_i curves shown in Figure S3. Black lines show the split-line regressions obtained by averaging the intercepts and slopes of each individual replicate. n=6 biological replicates are shown in different colours.

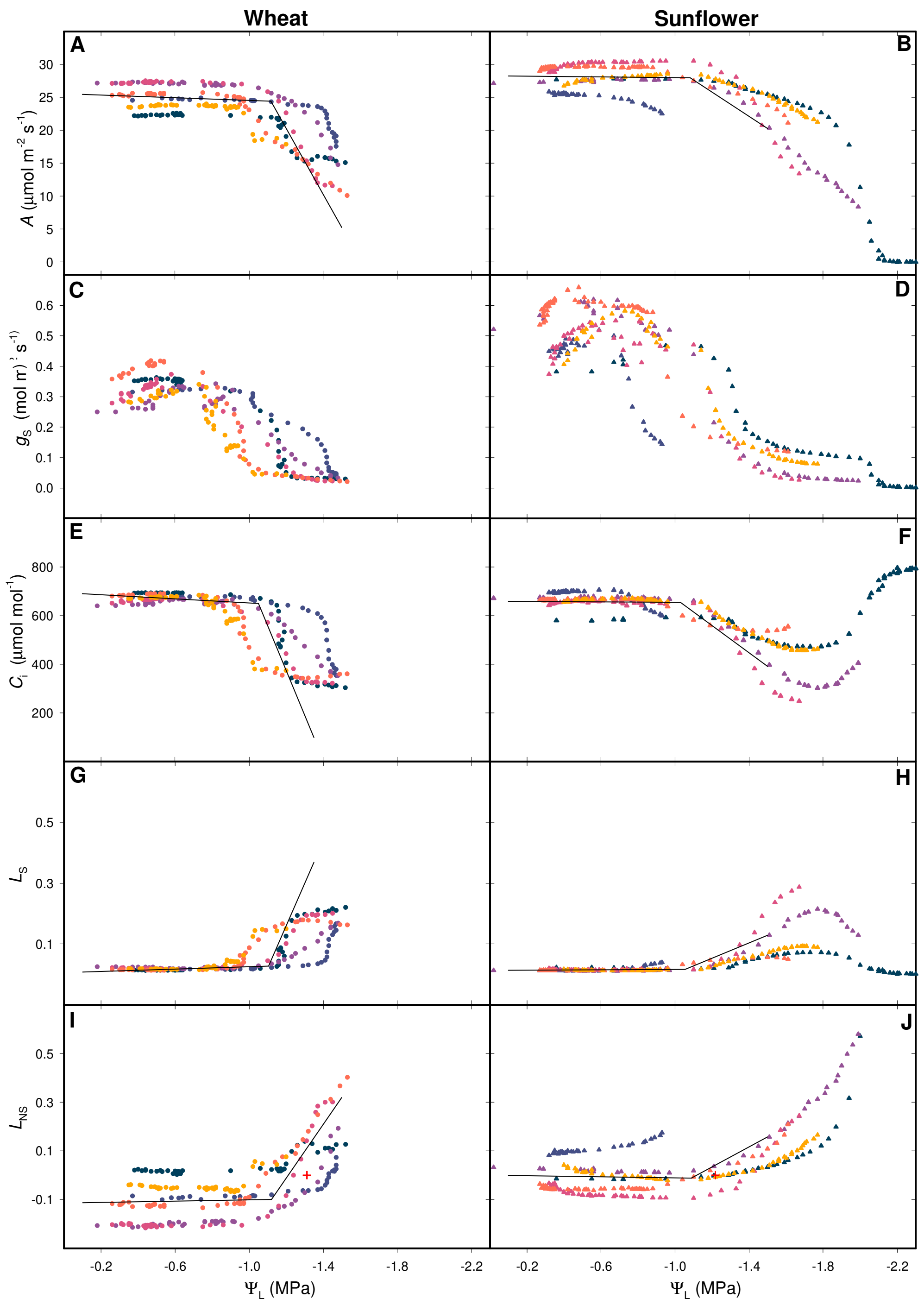

Figure S4. Concurrent gas exchange, fluorescence and isotopic discrimination

Wheat (left) and sunflower (right) plants grown on hydroponics were progressively pulled out of the water while water potential (Ψ_L) was measured at regular intervals, coupled with gas-exchange data, fluorescence, carbon and oxygen isotopic discrimination (Δ_o and (Δ_o^{18}), respectively), under ambient O_2 (closed symbols) or 2% O_2 (empty symbols). Panels **A** and **B**, yield of photosystem II (*Y(II)*); Panels **C** and **D**, Δ^{13}C; Panels **E** and **F** Δ^{18}O of CO_2. $n = 6$ biological replicates are shown in different colours.

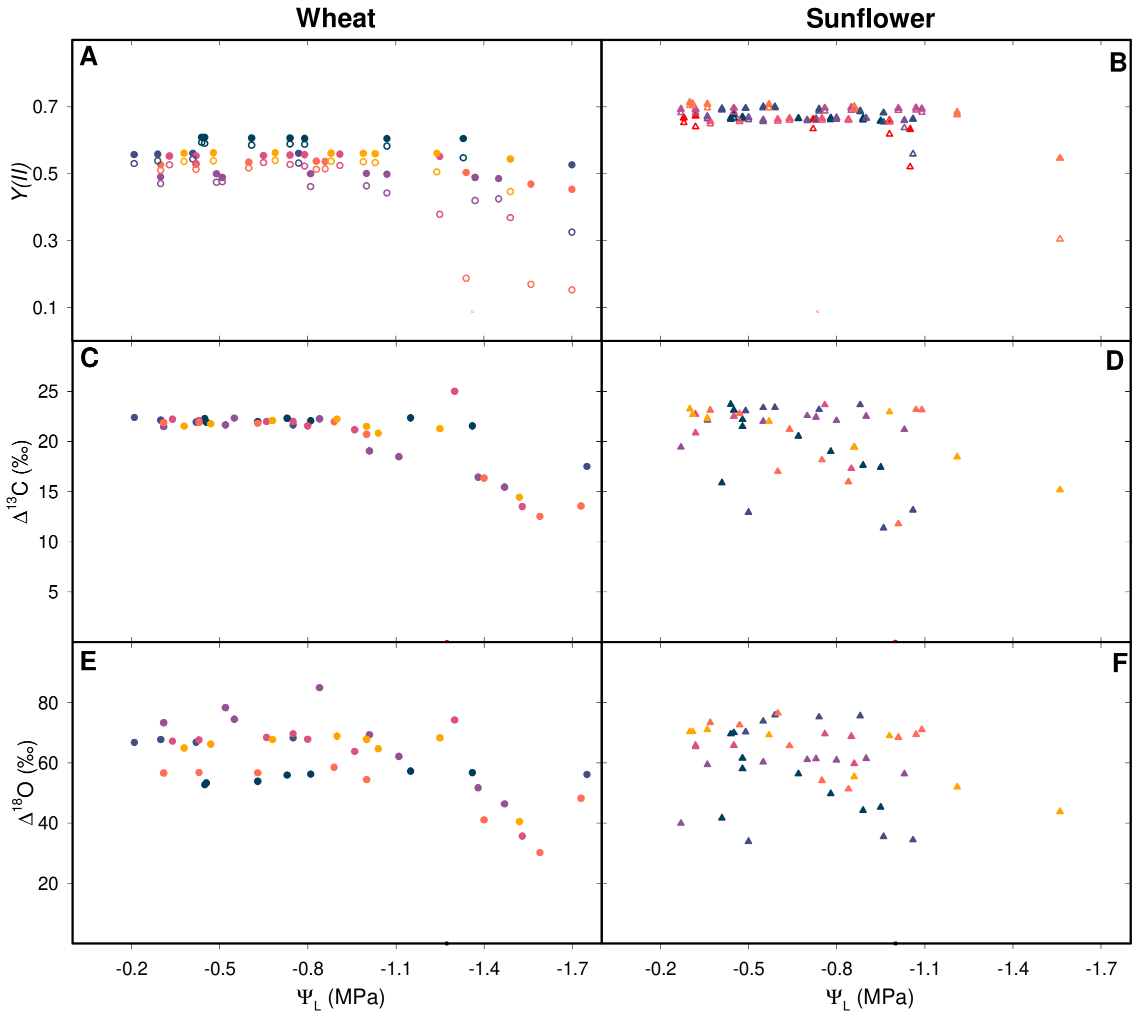

Figure S5. Relationship between net assimilation and mesophyll conductance.

Relationship between the net assimilation rate (A) and mesophyll conductance derived from chlorophyll fluorescence measurements ($^{J}g_{M}$), stable carbon isotope discrimination ($^{C}g_{M}$), and stable oxygen isotope discrimination ($^{O}g_{M}$).

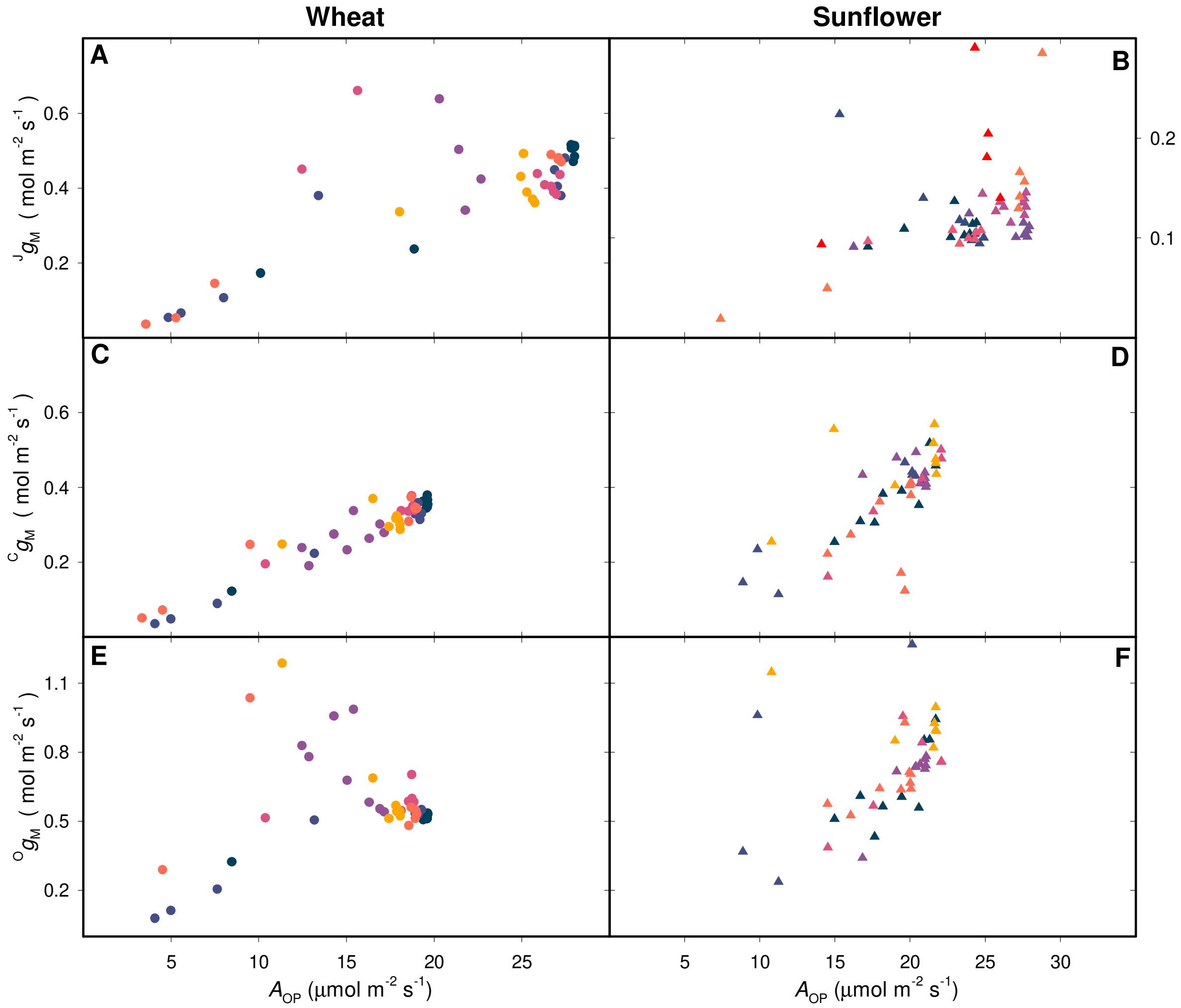

Figure S6. Sensitivity analysis of the effect of cuticular conductance, unsaturation and leaf temperature on estimates of mesophyll conductance in dehydrated leaves.

Relationship between variations in cuticular conductance (g_{cw}), relative humidity in the airspace (h_i) and systematic error in the measured leaf temperature (T_{leaf}) on g_M. The sensitivity analysis was conducted at the Ψ_L that resulted in approximately a 50% reduction in g_M, and expressed as relative to the g_M obtained under full hydration.

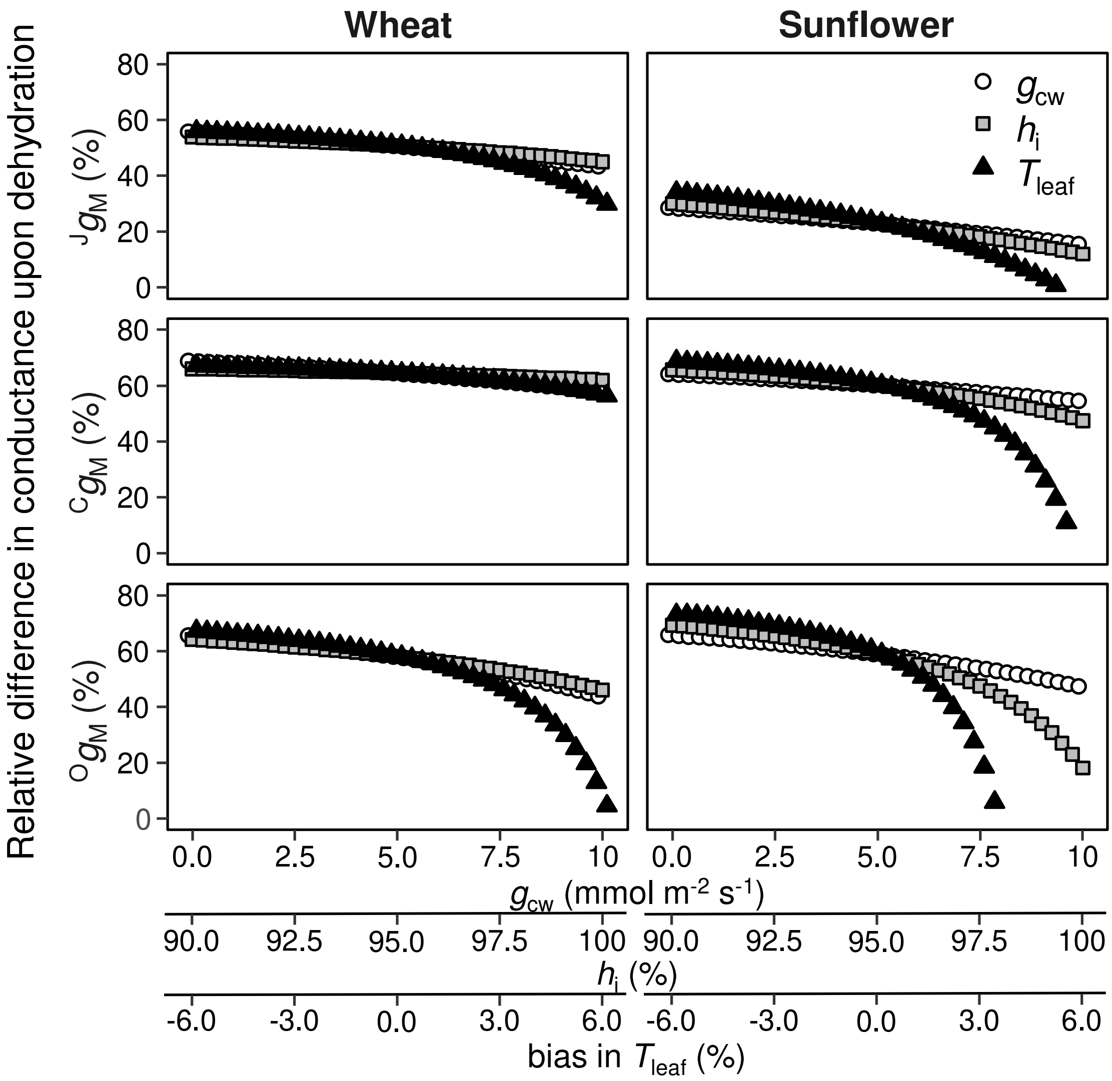

References

Bartlett MK, Scoffoni C, Sack L. 2012. The determinants of leaf turgor loss point and prediction of drought tolerance of species and biomes: a global meta-analysis. *Ecology Letters* **15**(5): 393-405.

Bellasio C. 2025. Quantifying photosynthetic restrictions. *Photosynthesis Research* **163**(2): 19.

Bellasio C, Beerling DJ, Griffiths H. 2016. An Excel tool for deriving key photosynthetic parameters from combined gas exchange and chlorophyll fluorescence: theory and practice. *Plant Cell and Environment* **39**(6): 1180–1197.

Bellasio C, Griffiths H. 2014. Acclimation to Low Light by C_4 maize: Implications for Bundle Sheath Leakiness. *Plant Cell and Environment* **37**(5): 1046-1058.

Bellasio C, Quirk J, Beerling DJ. 2018. Stomatal and non-stomatal limitations in savanna trees and C_4 grasses grown at low, ambient and high atmospheric CO_2. *Plant Science* **274**: 181-192.

Blackman CJ, Brodribb TJ, Jordan GJ. 2009. Leaf hydraulics and drought stress: response, recovery and survivorship in four woody temperate plant species. *Plant, Cell & Environment* **32**(11): 1584-1595.

Buckley TN, Farquhar GD, Mott KA. 1999. Carbon-water balance and patchy stomatal conductance. *Oecologia* **118**: 132-143.

Buckley TN, Sack L. 2019. The humidity inside leaves and why you should care: implications of unsaturation of leaf intercellular airspaces. *American Journal of Botany* **106**(5): 618-621.

Cernusak LA, Ubierna N, Jenkins MW, Garrity SR, Rahn T, Powers HH, Hanson DT, Sevanto S, Wong SC, McDowell NG. 2018. Unsaturation of vapour pressure inside leaves of two conifer species. *Scientific reports* **8**(1): 1-7.

Cernusak LA, Wong SC, Stuart‐Williams H, Márquez DA, Pontarin N, Farquhar GD. 2024. Unsaturation in the air spaces of leaves and its implications. *Plant, Cell & Environment*.

Cousins AB, Badger MR, Von Caemmerer S. 2006. Carbonic anhydrase and its influence on carbon isotope discrimination during C4 photosynthesis. Insights from antisense RNA in Flaveria bidentis. *Plant Physiology* **141**(1): 232-242.

Cousins AB, Badger MR, von Caemmerer S. 2008. C_4 photosynthetic isotope exchange in NAD-ME- and NADP-ME-type grasses. *Journal of Experimental Botany* **59**(7): 1695-1703.

Craig H. 1953. The Geochemistry of the Stable Carbon Isotopes. *Geochimica et Cosmochimica Acta* **3**(2-3): 53-92.

Diao H, Cernusak LA, Saurer M, Gessler A, Siegwolf RTW, Lehmann MM. 2024. Dry inside: progressive unsaturation within leaves with increasing vapour pressure deficit affects estimation of key leaf gas exchange parameters. *New Phytologist* **244**(4): 1275-1287.

Downton W, Loveys B, Grant W. 1988. Non‐uniform stomatal closure induced by water stress causes putative non‐stomatal inhibition of photosynthesis. *New Phytologist* **110**(4): 503-509.

Farquhar GD. 1983. On the Nature of Carbon Isotope Discrimination in C_4 Species. *Australian Journal of Plant Physiology* **10**(2): 205-226.

Farquhar GD, Cernusak LA. 2012. Ternary effects on the gas exchange of isotopologues of carbon dioxide. *Plant Cell and Environment* **35**(7): 1221-1231.

Farquhar GD, Lloyd J 1993. Carbon and oxygen isotope effects in the exchange of carbon dioxide between terrestrial plants and the athmosphere. In: Ehleringer JR ed. *Stable Isotopes and Plant Carbon - Water relations*. New York: Academic Press, 47-70.

Farquhar GD, Walker DA, Osmond CB. 1989. Models of integrated photosynthesis of cells and leaves. *Philosophical Transactions of the Royal Society of London. B, Biological Sciences* **323**(1216): 357-367.

Garen JC, Branch HA, Borrego I, Blonder B, Stinziano JR, Michaletz ST. 2022. Gas exchange analysers exhibit large measurement error driven by internal thermal gradients. *New Phytol* **236**(2): 369-384.

Genty B, Briantais JM, Baker NR. 1989. The relationship between the quantum yield of photosynthetic electron-transport and quenching of chlorophyll fluorescence. *Biochimica Et Biophysica Acta* **990**(1): 87-92.

Gillon JS, Yakir D. 2000a. Internal conductance to CO_2 diffusion and $CO^{18}O$ discrimination in C_3 leaves. *Plant Physiology* **123**(1): 201-213.

Gillon JS, Yakir D. 2000b. Naturally low carbonic anhydrase activity in C_4 and C_3 plants limits discrimination against (COO)-O-18 during photosynthesis. *Plant Cell and Environment* **23**(9): 903-915.

Holloway-Phillips M, Cernusak LA, Stuart-Williams H, Ubierna N, Farquhar GD. 2019. Two-source δ18O method to validate the CO18O-photosynthetic discrimination model: implications for mesophyll conductance. *Plant Physiology* **181**(3): 1175-1190.

Jain P, Huber AE, Rockwell FE, Sen S, Holbrook NM, Stroock AD. 2024. New approaches to dissect leaf hydraulics reveal large gradients in living tissues of tomato leaves. *New Phytologist* **242**(2): 453-465.

Laisk A. 1983. Calculation of Leaf Photosynthetic Parameters Considering the Statistical Distribution of Stomatal Apertures. *Journal of Experimental Botany* **34**(12): 1627-1635.

Lanigan GJ, Betson N, Griffiths H, Seibt U. 2008. Carbon Isotope Fractionation during Photorespiration and Carboxylation in Senecio. *Plant Physiology* **148**(4): 2013-2020.

Márquez DA, Stuart-Williams H, Cernusak LA, Farquhar GD. 2023. Assessing the CO_2 concentration at the surface of photosynthetic mesophyll cells. *New Phytologist* **238**(4): 1446-1460.

Márquez DA, Stuart-Williams H, Farquhar GD. 2021. An improved theory for calculating leaf gas exchange more precisely accounting for small fluxes. *Nature Plants* **7**(3): 317-326.

Márquez DA, Stuart-Williams H, Farquhar GD, Busch FA. 2022. Cuticular conductance of adaxial and abaxial leaf surfaces and its relation to minimum leaf surface conductance. *New Phytologist* **233**(1): 156-168.
McNevin DB, Badger MR, Kane HJ, Farquhar GD. 2006. Measurement of (carbon) kinetic isotope effect by Rayleigh fractionation using membrane inlet mass spectrometry for CO_2-consuming reactions. *Functional Plant Biology* **33**(12): 1115-1128.
Mott KA, Buckley TN. 1998. Stomatal heterogeneity. *Journal of Experimental Botany*: 407-417.
Mott KA, Buckley TN. 2000. Patchy stomatal conductance: emergent collective behaviour of stomata. *Trends in Plant Science* **5**(6): 258-262.
Mott KA, Peak D. 2011. Alternative perspective on the control of transpiration by radiation. *Proceedings of the National Academy of Sciences* **108**(49): 19820-19823.
O'Leary MH. 1984. Measurement of the isotope fractionation associated with diffusion of carbon dioxide in aqueous solution. *The Journal of Physical Chemistry* **88**(4): 823-825.
Ogée J, Wingate L, Genty B. 2018. Estimating mesophyll conductance from measurements of $C^{18}OO$ photosynthetic discrimination and carbonic anhydrase activity. *Plant Physiology* **178**(2): 728-752.
Pospíšilová J, Šantrůček J. 1994. Stomatal patchiness. *Biologia Plantarum* **36**: 481-510.
Prioul JL, Chartier P. 1977. Partitioning of Transfer and Carboxylation Components of Intracellular Resistance to Photosynthetic CO_2 Fixation: A Critical Analysis of the Methods Used. *Annals of Botany* **41**(4): 789-800.
Quirk J, Bellasio C, Johnson DA, Beerling DJ. 2019. Response of photosynthesis, growth and water relations of a savannah-adapted tree and grass grown across high to low CO_2. *Annals of Botany* **124**(1): 77-90.
Rockwell FE. 2024. Shorting the metaphorical circuit: vascular partitioning and stomatal patchiness can create apparent unsaturation and CO_2 gradient inversion in the Ohmic analogy for leaf gas exchange. *New Phytologist* **244**(5): 1812-1823.
Rockwell FE, Holbrook NM, Jain P, Huber AE, Sen S, Stroock AD. 2022. Extreme undersaturation in the intercellular airspace of leaves: a failure of Gaastra or Ohm? *Annals of Botany* **130**(3): 301-316.
Roeske CA, Oleary MH. 1984. Carbon Isotope Effects on the Enzyme-Catalyzed Carboxylation of Ribulose Bisphosphate. *Biochemistry* **23**(25): 6275-6284.
Slot M, Nardwattanawong T, Hernández GG, Bueno A, Riederer M, Winter K. 2021. Large differences in leaf cuticle conductance and its temperature response among 24 tropical tree species from across a rainfall gradient. *New Phytologist* **232**(4): 1618-1631.
Terashima I, Wong SC, Osmond CB, Farquhar GD. 1988. Characterization of Non-Uniform Photosynthesis Induced by Abscisic-Acid in Leaves Having Different Mesophyll Anatomies. *Plant and Cell Physiology* **29**(3): 385-394.
Tholen D. 2024. GasanalyzeR: Advancing Reproducible Research using a New R Package for Photosynthesis Data Workflows. *AoB Plants*: plae035.
Trueba S, Pan R, Scoffoni C, John GP, Davis SD, Sack L. 2019. Thresholds for leaf damage due to dehydration: declines of hydraulic function, stomatal conductance and cellular integrity precede those for photochemistry. *New Phytologist* **223**(1): 134-149.
Ubierna N, Gandin A, Boyd RA, Cousins AB. 2017. Temperature response of mesophyll conductance in three C4 species calculated with two methods: ^{18}O discrimination and in vitro Vpmax. *New Phytologist* **214**(1): 66–80.
Williams TG, Flanagan LB, Coleman JR. 1996. Photosynthetic gas exchange and discrimination against $^{13}CO_2$ and $C^{18}O^{16}O$ in tobacco plants modified by an antisense construct to have low chloroplastic carbonic anhydrase. *Plant Physiology* **112**(1): 319-326.
Wong SC, Canny MJ, Holloway-Phillips M, Stuart-Williams H, Cernusak LA, Márquez DA, Farquhar GD. 2022. Humidity gradients in the air spaces of leaves. *Nature Plants* **8**(8): 971-978.
